# Highly Synergistic Effects of Melittin With Vancomycin and Rifampin Against Vancomycin and Rifampin Resistant *Staphylococcus epidermidis*

**DOI:** 10.3389/fmicb.2022.869650

**Published:** 2022-06-23

**Authors:** Rasoul Mirzaei, Mohammad Yousef Alikhani, Carla Renata Arciola, Iraj Sedighi, GholamReza Irajian, Elaheh Jamasbi, Rasoul Yousefimashouf, Kamran Pooshang Bagheri

**Affiliations:** ^1^Department of Microbiology, School of Medicine, Hamadan University of Medical Sciences, Hamadan, Iran; ^2^Venom and Biotherapeutics Molecules Laboratory, Medical Biotechnology Department, Biotechnology Research Center, Pasteur Institute of Iran, Tehran, Iran; ^3^Laboratorio di Patologia delle Infezioni Associate all’Impianto, IRCCS Istituto Ortopedico Rizzoli, Bologna, Italy; ^4^Laboratorio di Immunoreumatologia e Rigenerazione Tissutale, IRCCS Istituto Ortopedico Rizzoli, Bologn, Italy; ^5^Department of Experimental, Diagnostic and Specialty Medicine, University of Bologna, Bologna, Italy; ^6^Department of Pediatrics, School of Medicine, Hamadan University of Medical Sciences, Hamadan, Iran; ^7^Microbial Biotechnology Research Center, Iran University of Medical Sciences, Tehran, Iran; ^8^Department of Microbiology, School of Medicine, Iran University of Medical Sciences, Tehran, Iran; ^9^Research Center of Oils and Fats, Kermanshah University of Medical Science, Kermanshah, Iran

**Keywords:** MRSE, MDR, rifampin-resistant, vancomycin-resistant, melittin, synergism

## Abstract

Methicillin-resistant *Staphylococcus epidermidis* (MRSE) strains are increasingly emerging as serious pathogens because they can be resistant to many antibiotics called multidrug resistance (MDR) that limit the therapeutic options. In the case of vancomycin- and rifampin-resistant MDR-MRSE, the physicians are not allowed to increase the doses of antibiotics because of severe toxicity. Accordingly, we investigated the synergistic activity of melittin antimicrobial peptide with vancomycin and rifampin against vancomycin-resistant, and rifampin-resistant MDR-MRSE isolates. Minimum inhibitory concentration (MIC), minimum bactericidal concentration (MBC), fractional inhibitory concentration index (FICi), and fractional bactericidal concentration index (FBCi) of antimicrobial agents against isolates were determined. Coagulate activities and serum and salt stability as well as melittin cytotoxicity on the human embryonic kidney (HEK) 293 cells and human red blood cells (RBCs) at their synergistic concentrations. MIC and MBC values for melittin were in the range of 0.312–2.5 and 0.312–5, respectively. Results also showed that the interaction of melittin with drugs was highly synergistic in which the geometric means of FICi and FBCi were < 0.5. Induced synergism led to a decrease in melittin, rifampin, and vancomycin concentrations by 8–1,020, 2–16, and 4–16-folds, respectively. This phenomenon caused a reduction in melittin toxicity by which the synergistic concentration of melittin needed to kill bacteria did not show cytotoxicity and hemolytic activity. Besides, no coagulation activity was found for the synergistic and alone concentrations of melittin in both Prothrombin Time (PT) and Partial Thromboplastin Time (PTT). Interestingly, the antibacterial activity of melittin in Mueller Hinton Broth (MHB) containing human serum did no significant differences between MIC and MBC values of melittin in MHB and MHB containing 10% human serum. The present findings showed that the therapeutic index of melittin was improved by 32.08- and 12.82-folds when combined with vancomycin and rifampin, respectively. Taken together, the obtained data show that melittin alone was effective against MDR-MRSE isolates and this antimicrobial peptide showed highly synergistic effects with vancomycin and rifampin without causing toxicity. Therefore, the combination of melittin and traditional antibiotics could be a promising strategy for the treatment of infections caused by MDR-MRSE.

## Introduction

*Staphylococcus epidermidis* is a permanent part of the skin and mucous membranes microbiota which, by adhering to human tissue components by specific adhesins, could establish a prolonged commensal communication with humans (Otto, 2009). Currently, with the increase of indwelling medical device applications, this bacterium has been emerged as a crucial opportunistic pathogen responsible for nosocomial and implant-related infections, particularly in immune-compromised and hospitalized patients (Otto, 2009; Rasoul et al., 2019). Noticeably, *S. epidermidis* belongs to the bacterial species reported to be at first place (or second place after *Staphylococcus aureus*) of etiological agents of causing infections in orthopedic implants (Arciola et al., 2002, 2006). Moreover, *S. epidermidis* is well-known for its wide antimicrobial resistance and the ability to form biofilms, resulting in difficulty of treatment in *S. epidermidis* associated infections (Gómez-Sanz et al., 2019; Mirzaei et al., 2022). In this regard, methicillin-resistant *S. epidermidis* (MRSE) strains have emerged as infectious pathogens as they are commonly endowed with additional antibiotic resistance (Cherifi et al., 2013; Sabaté Brescó et al., 2017). Of note, this ability could provide a risk for the transfer of drug- resistance toward highly pathogenic bacterium, *S. aureus* (Bloemendaal et al., 2010). On top of this, nowadays, multidrug-resistant MRSE (MDR-MRSE) strains drastically limit the available therapeutic options and represent a crucial challenge for human health (Miragaia et al., 2007; Ibrahem et al., 2008; Li et al., 2009).

The antibiotic of choice for MDR-MRSE infections is glycopeptides, such as vancomycin. Also, the addition of rifampin has been shown to enhance therapeutic indices, particularly against slow-growing organisms in chronic biofilm infections (Eom and Kim, 2001). Nevertheless, in current decades, vancomycin-resistant *S. epidermidis* (VRSE), as well as rifampin-resistant *S. epidermidis* infections were reported over the world (Cherubin et al., 1981; Tuazon and Miller, 1983; Sanyal et al., 1991; Krcmery et al., 1996; Zimmerli et al., 1998, 2004). In this regard, bacterial resistance commonly develops with monotherapy with either agent; hence, these antibiotics should always be administrated in combination with other effective agents (Howden et al., 2010). Antibiotic therapy alone often fails to treat MDR-MRSE infections, and the removal of medical devices may be the only way. Besides, vancomycin-induced nephrotoxicity and ototoxicity at high serum concentrations urge the careful monitoring of vancomycin in patients (Ong and Nicolau, 2004). It has been described that vancomycin at plasma levels higher than 20 μg/mL is related to nephrotoxicity (Barceló-Vidal et al., 2018). Rifampicin has been found to cause renal, hepatic, hematological disorders, and convulsions (Sridhar et al., 2012). Rifampin should also be used in combination with other appropriate antimicrobial agents to prevent the emergence of resistant strains during the treatment of infectious pathogens (Ju et al., 2006; Gidari et al., 2020).

Accordingly, it has been found that the combination of antimicrobial peptides (AMPs) with conventional antibiotics can be a promising strategy against MDR bacterial pathogens (Zusman et al., 2013; Schmid et al., 2019). Currently, AMPs are found as novel developing antibacterial agents that could serve as an alternative to antibiotics (Bardbari et al., 2018; Memariani et al., 2018; Aghazadeh et al., 2019; Akbari et al., 2019; Bevalian et al., 2021; Zarghami et al., 2021a). Furthermore, the application of AMPs along with conventional antibiotics usually enhances the results of mono drug therapies (Bardbari et al., 2018; Akbari et al., 2019). Melittin as an amphipathic, alpha-helical, and cationic AMP (Dezfuli et al., 2014) could act against a wide variety of Gram-positive, and Gram-negative pathogens (Choi et al., 2015; Karaaslan et al., 2016; Khozani et al., 2019; Memariani et al., 2019; Pashaei et al., 2019; Zarghami et al., 2021b). It has been found that this highly potent antibacterial peptide has acceptable synergistic effects on the killing of MDR pathogens (Jamasbi et al., 2016; Akbari et al., 2019). Hence, in the present study, we aimed to assess the effects of melittin alone and in combination with vancomycin and rifampin against vancomycin-resistant and rifampin-resistant MDR-MRSE isolates.

## Materials and Methods

### Media, Chemical Reagents, and Antibiotics

All the antibiotic disks were purchased from MAST Company (United Kingdom). Vancomycin and rifampin powders were obtained from Sigma-Aldrich [Saint Louis (St. Louis), Missouri (MO), United States]. Mannitol Salt Agar, Blood Agar, DNA agar, Mueller-Hinton Agar (MHA), Mueller-Hinton broth (MHB), NaCl, and MgCl_2_ were purchased from Merck (Merck Company, United States). The human embryonic kidney cells (HEK-293) were kindly provided by Dr. Ali Teimoori (Hamadan, Iran). The Dulbecco’s Modified Eagle’s Medium (DMEM), 3-(4, 5-dimethyl-2-thiazolyl)-2, 5-diphenyl-2 H-tetrazolium bromide (MTT), fetal bovine serum (FBS), and dimethyl sulfoxide (DMSO) were purchased from Sigma-Aldrich (St. Louis, MO, United States). *S. epidermidis* ATCC (American Type Culture Collection) 35984 was kindly provided by Dr. Eyup Dogan (Biotechnology Institute, Ankara, Turkey) and Dr. Fereshteh Saffari (Kerman University of Medical Sciences, Kerman, Iran). *S. epidermidis* DSMZ (Deutsche Sammlung von Mikroorganismen und Zellkulturen) 3,270 was kindly provided by Prof. Bibi Sedigheh Fazly Bazzaz (Mashhad University of Medical Sciences, Mashhad, Iran). *S. epidermidis* ATCC 12228, *S. aureus* ATCC 25923, and ATCC 29213 were purchased from the Pasteur Culture Collection of Tehran, Iran.

### Peptide Synthesis

Melittin (GIGAVLKVLTTGLPALISWIKRKRQQH2) was synthesized in > 96% purity using the solid-phase synthesis technique (DGpeptides Co., Ltd., China). The purity of the synthetic peptides was analyzed *via* reversed-phase high-performance liquid chromatography that was performed by the DGpeptides company. The accuracy of synthesis was controlled by mass spectrometry on a liquid chromatography-mass spectrometry (LC-MS) instrument by the DGpeptides company. The peptide concentration and its purity were reconfirmed using bicinchoninic acid assay (BCA) and Reversed-phase high-performance liquid chromatography (RP-HPLC), respectively, as described earlier (Eisapoor et al., 2016).

### Collection, and Confirmation of *Staphylococcus epidermidis* Isolates

From April 2019 to August 2019, 97 *S. epidermidis* isolates were collected from hospitalized patients at various wards from different hospitals in Hamedan, Iran. The isolates were taken from patients of both gender and various age ranges. Also, the isolates were not duplicated, and only one sample was obtained from each patient. The *S. epidermidis* isolates were isolated from blood (*n* = 46), catheter (*n* = 17), wound (*n* = 15), urine (*n* = 14), and sputum (*n* = 5). In the present study, the inclusion criteria were in line with guidelines established by the Center for Disease Control (Atlanta, United States) (Horan et al., 2008). Briefly, all isolates were directly cultured on Blood Agar at 37 ^°^ C for 24 h, and then *S. epidermidis* isolates were characterized using Gram staining, catalase reaction, coagulase test, colony morphology, resistance to bacitracin disks and polymyxin B, sensitivity to novobiocin disk, negative-mannitol fermentation, and -DNAase activity (Murray et al., 2009; Talebi et al., 2016).

### Susceptibility Assays for Isolates

In the present study, for the detection of MRSE, the disk diffusion method was carried out. Finally, antibacterial susceptibility patterns of chosen isolates were determined based on disk diffusion, minimum inhibitory concentration (MIC), and minimum bactericidal concentration (MBC).

#### Disk Diffusion

*Staphylococcus epidermidis* isolates were monitored in terms of their resistance to methicillin by cefoxitin (30 μg), and oxacillin (1 μg) *via* disk diffusion method according to the Clinical and Laboratory Standards Institute (CLSI) recommendations (Wayne, 2010). In brief, fresh bacterial isolates were grown in the MHB at 37°C overnight with shaking at 180 rpm, and the next day, the number of mid-logarithmic bacterial cells was adjusted to the 0.5 McFarland turbidity standard. In this regard, the optical density (OD) that is conventionally in a range between 0.08 and 0.1 [equal to 10^8^ Colony-forming units (CFUs)/mL], was set to 0.09 in the current study to enhance the accuracy of cell quantification. Then, the suspension 0.5 McFarland standard was swabbed onto the Mueller–Hinton agar plates, and antibiotic disks were placed on top of the agar plates. The plates were incubated at 37°C for 18–24 h based on examined agents. After incubation, the size of the inhibition zone formed around each disk was measured. Finally, 20 isolates were selected for further evaluation.

To determine the antibiotic susceptibility pattern of selected isolates, as mentioned earlier, the disk diffusion method was conducted based on the CLSI recommendations for the following disks: tetracycline (30 μg), clindamycin (2 μg), gentamicin (10 μg), trimethoprim-sulfamethoxazole (1.25 μg), erythromycin (15 μg), penicillin (10 units), and linezolid (30 μg). The *S. aureus* ATCC 25923 strain was applied as the control strain (Wayne, 2010). In addition, MDR isolates were characterized by observing the resistance against at least one or more antibiotics in three or more classes of antibiotics (Magiorakos et al., 2012).

#### Minimum Inhibitory Concentration

The broth microdilution assay was performed according to the CLSI recommendations to survey the MIC values of melittin, vancomycin, and rifampin in selected *S. epidermidis* isolates (Wayne, 2010). In summary, fresh bacterial isolates were grown in the MHB medium at 37°C overnight with shaking at 180 rpm, and the next day, the number of mid-logarithmic bacterial cells was adjusted to 0.5 McFarland turbidity standard. In this regard, the OD which is conventionally in a range between 0.08 and 0.1 (equal to 10 ^8^ CFUs/mL), was set at 0.09 in the current study to enhance the accuracy of cell quantification, and then the numbers of bacterial cells were adjusted to 10^6^ CFUs/mL quickly in the same medium. Besides, at the same time, two-fold serial dilutions of antibiotics and melittin were prepared in the same medium at a volume of 100 μL in a 96-well flat-bottom microplate (Jet Biofil, Guangzhou, China). The melittin, vancomycin, and rifampin ranged from 0.0391–20 μg, 0.25–128 μg, and 0.00075–1,024 μg, respectively. Finally, 100 μl of the bacterial stock containing 10 ^5^ CFUs/mL was inoculated into each well of the serially diluted melittin or antibiotics, and the microplates were incubated at 37°C for 18–24 h based on examined agents. For the determination of MIC, based on the CLSI definition, the lowest value of the examined agents that completely inhibited the visible bacterial growth was considered (Wayne, 2010). The MIC experiments were repeated three times for all isolates.

#### Minimum Bactericidal Concentration and Determination of MBC/MIC Ratio

The MBC values were measured for rifampin, vancomycin, and melittin according to the CLSI recommendations (Wayne, 2010). In brief, fresh bacterial isolates were grown in the MHB medium at 37°C overnight with shaking at 180 rpm, and the next day the number of mid-logarithmic bacterial cells was adjusted to the 0.5 McFarland turbidity standard, and then 10 ^6^ CFUs/mL, as mentioned above. Besides, at the same time, two-fold serial dilutions of vancomycin, rifampin, and melittin were prepared in the same medium at a volume of 100 μL in a 96-well flat-bottom microplate (Jet Biofil, Guangzhou, China). Finally, 100 μl of the bacterial stock containing 10^5^ CFUs/mL was inoculated into each well of the serially diluted melittin or antibiotics, and then the microplates were incubated at 37°C for 24 h. Afterward, to determine the MBC values, after 24 h of incubation, 10 μL of each well was cultured on the MHA medium and the grown colonies were completely counted. The MBC values of melittin, vancomycin, and rifampin were considered as the lowest amount of agents required to kill 100% of the cultured isolates.

Finally, the MBC/MIC ratio was calculated to characterize the existence/absence of antimicrobial agent tolerance in isolates. The antimicrobial agent tolerance was defined by an MBC/MIC ratio of ≥ 32 and/or an MBC/MIC ratio of ≥ 16 when the MBC value was ≥ to the resistance breakpoint offered by CLSI (Traczewski et al., 2009).

### Measurement of Synergistic Effects

The synergistic effects of melittin, vancomycin, and rifampin were assessed using broth microdilution checkerboard with major modifications based on MIC values for 6 selected *S. epidermidis* isolates, including MDR-MRSE 1, Non-MDR-MRSE 4, MDR-MRSE 5, Non-MDR-MSSE 1, MDR-MRSE 8, and *S. epidermidis* ATCC 35984.

Briefly, the checkerboard microdilution method was performed according to the obtained MIC values with major modifications as follows: dilutions of each of melittin (from 5 to 0.0049 μg), rifampin (from 256 to 0.03125 μg), and vancomycin (from 32 to 0.0125 μg) were added to the wells of 96-well flat-bottom microplates (Jet Biofil, Guangzhou, China) at a volume of 100 μL, and at the same time, the bacterial stock was prepared, as mentioned earlier. Then, 100 μL of the diluted bacterial stock containing 10^5^ CFUs/mL was added to each well and the microplate was incubated at 37°C for 24 h. Afterward, the lowest value of the examined agents that completely inhibited the visible bacterial growth was considered, and in the following step, the fractional inhibitory concentration index (FICi) of the two combined anti-bacterial agents was calculated as follows: FIC = (MIC drug A in combination/MIC drug A alone) + (MIC drug B in combination/MIC drug B alone). FIC indices pointed to the type of drug interaction if the following data are established: Synergy, values *n* ≤ 0.5; Partial synergy, values 0.5 < *n* < 1; Additive effect, for a value *n* = 1; Indifferent effect, for values 1 < *n* < 4; Antagonistic effect, for a value 4 ≤ *n* (Akbari et al., 2019).

Most importantly, it should be noted that we used broth microdilution checkerboard with major modifications based on the MBC values, so-called fractional bactericidal concentration index (FBCi) to detect interactions between antibacterial agents based on the MBC values. Briefly, the antibacterial agents-interaction method was carried out similar to the method used for FIC, and, at the same time, the bacterial stock was prepared as previously mentioned, and then, 100 μL of the diluted bacterial stock containing 10^5^ CFUs/mL was added to each well and the microplate was incubated at 37°C for 24 h. After that, 10 μL from each well was cultured on MHA and the MBC values of melittin, vancomycin, and rifampin were characterized as the lowest concentration of agent required to kill 100% of the cultured isolates. Finally, FBCi was calculated as follows: FBCi = (MBC drug A in combination/MBC drug A alone) + (MBC drug B in combination/MBC drug B alone). FBC indices pointed to the type of antibacterial agents’ interaction as mentioned for FICi (Mackay et al., 2000).

### Serum, and Salt Stability Testing for Melittin

To survey the stability of melittin for possible clinical trials, the antibacterial activity of this peptide after encounter to serum, and salt against selected strains was determined (Memariani et al., 2016). In this regard, mid-logarithmic bacterial strains diluted to 10^5^ CFUs/mL as above mentioned in MHB, added to MHB containing 10% human serum, and/or 150 mM NaCl and 1 mM MgCl_2,_ and incubated at 37°C for 2 h (Memariani et al., 2016). Afterward, the MIC and MBC of melittin against strains were again determined as above mentioned.

### *In vitro* Toxicity Assay

To confirm the safe administration of melittin for possible future clinical trials, cytotoxicity of melittin at the synergistic concentrations was assessed by MTT. Besides, to monitor the effect of the entry of melittin into the bloodstream, the hemolysis rate of human Red Blood Cells (RBCs) was assessed at the synergistic concentrations. Finally, coagulation effects of melittin at the synergistic concentrations was assessed by prothrombin time (PT) and partial thromboplastin time (PTT).

#### MTT Assay for Melittin

The cytotoxicity of melittin (at the MIC, MBC, and the best selected synergistic concentrations) was assessed by MTT assay, as previously described (Akbari et al., 2019). In brief, the HEK-293 cells were cultured in the DMEM medium supplemented with 10% Fetal-Calf Serum (FCS) and antibiotics (100 U/mL streptomycin, and 100 U/mL penicillin). Then, the cells were with 5% CO_2_ and 95% humidity incubated at 37°C. In brief, the cells were seeded at a density of 4 × 10^4^ cells/well and incubated for 24 h. Melittin at concentrations of 0.004, 0.009, 0.019, 0.039, 0.156, 2.5, and 5 μg was added to the wells of microplate and incubated at 37°C for 24 h. Next, 20 μL of MTT stock solution (5 mg/ml) was added to each well and incubated for 4 h. The supernatants were then discarded followed by the addition of 100 μL of DMSO to the wells. The absorbance of the wells was measured at a wavelength of 570 nm using a microplate spectrophotometer (Synergy™ HTX Multi-Mode Microplate Reader-BioTek Co., United States). All experiments were conducted in triplicate. The percentage of cell survival was calculated according to the following formula:


(1) S⁢u⁢r⁢v⁢i⁢v⁢a⁢l⁢p⁢e⁢r⁢c⁢e⁢n⁢t=(O⁢D⁢t⁢e⁢s⁢t/O⁢D⁢c⁢o⁢n⁢t⁢r⁢o⁢l)×100

#### Hemolytic Activity of Melittin

For the survey of hemolytic activity, melittin at the MIC, MBC, and the best selected synergistic concentrations was used according to the previously described method (Zarrinnahad et al., 2018). In summary, human RBCs (hRBCs) were obtained from an individual participant, who signed the informed consent for venipuncture procedure, into a glass tube containing heparin and harvested by centrifugation. After separation, 200 μl of packed erythrocytes were re-dispersed in 9.8 ml of PBS (0.08 M NaCl, 0.043 M Na2HPO4, 0.011 M KH2PO4), at pH 7.4 to get a 2% suspension of erythrocytes. The fresh hRBCs were rinsed thrice and resuspended in PBS (pH 7.4) to yield a 2% (v/v) erythrocytes/PBS suspension. In this regard, heparinized blood from a healthy volunteer was collected, centrifuged at 3,500 × rpm for 10 min, and washed with PBS (0.08 M NaCl, 0.043 M Na2HPO4, 0.011 M KH2PO4) three times, and the supernatant was discarded and 2% RBCs suspension prepared with PBS, and then 100 μL of this 2% RBC suspension was transferred to a 96-well microplate.

Then melittin at concentrations of 0.004, 0.009, 0.019, 0.039, 0.156, 2.5, and 5 μg was added to the RBCs, and the microplate was incubated at 37°C for 2 h and then centrifuged at 3,000 × rpm for 10 min. One hundred μl of supernatant from each well was moved gently to a new 96-well microplate and the OD of liberated hemoglobin was detected at 540 nm with a microplate spectrophotometer (Synergy™ HTX Multi-Mode Microplate Reader-BioTek Co., United States). The results were compared with the positive control (100 μL RBC and 100 μL Triton X-100 1%) and negative control (100 μL RBC and 100 μL PBS), and all experiments were performed in triplicate. Finally, the percent of hemolysis for melittin was determined by the following formula: [(OD sample-OD negative control)/(OD positive control-OD negative control)] × 100(Zarrinnahad et al., 2018).

#### Determination of Coagulation Effects on Human Plasma

Effect of melittin alone and in synergistic concentrations with vancomycin and rifampin on extrinsic and intrinsic pathways of coagulation were evaluated in human plasma by PT and partial PTT, respectively. To confirm the health situations of the donor, PT and PTT assays were performed and controlled with standard control reagent recommended by the manufacturer (Fisher Scientific Co., United States).

Briefly, PT assay was done as follow: Plasma was collected from a healthy donor, and different amounts of melittin (0.004, 0.009, 0.019, 0.039, 0.156, 2.5, and 5 μg) were added to citrated plasma (100 μL) and incubated for 5 min at 37°C in a water bath. Thromboplastin-D (200 μL) was then added, and clotting time was registered by a digital timer. Citrated plasma and PT control reagent were used as normal control. Additionally, PTT assay was done as follows: different amounts of melittin (0.004, 0.009, 0.019, 0.039, 0.156, 2.5, and 5 μg) were added to 100 μL of citrated plasma and incubated for 5 min at 37°C. One hundred μL of a PTT reagent was then added and mix, and then 100 μL of 0.1 M calcium chloride was added and clotting time was registered by a digital timer. Citrated plasma and PTT control reagent were also used as a control.

### Calculation of Therapeutic Index of Melittin

The therapeutic index was calculated according to Memariani et al. (Chen et al., 2005) with major modification as the ratio of the minimum hemolytic concentration to MBC of melittin alone or combination with antibiotics.

### Statistical Analysis

The GraphPad Prism (version 9) was used for the statistical analyses. In this regard, a paired-sample *T*-test was applied to analyze the statistical significance between melittin and melittin-antibiotic combinations in terms of antibacterial activities, as well as between the FIC and FBC indices. Besides, the one-way ANOVA was utilized to compare the differences in survival percent of the HEK-293 between various concentrations of melittin and the control. Quantitative data of the MIC and MBC in each antimicrobial agent were statistically represented as a minimum, maximum, median, and range. The results are generally expressed as the means and standard deviation (SD) unless otherwise indicated. All the statistical analyses were performed at a CI of 95%, and the statistical significance was set at the *p*-value < 0.05.

## Results

### MRSE Isolates

In the present study, the result of the antibacterial susceptibility pattern of *S. epidermidis* isolates toward cefoxitin, and oxacillin showed that 72.1% (*n* = 70) of *S. epidermidis* isolates were detected as MRSE. In particular, 78.2% (*n* = 36), 73.3% (*n* = 11), 64.2% (*n* = 9), 64.7% (*n* = 11), and 60% (*n* = 3) of blood, wound, urine, catheter, and sputum derived isolates were methicillin-resistant, respectively.

### Disk Diffusion, and MDR *S. epidermidis* Isolates

According to the result of the disk diffusion method, the antibiotic resistance rate of *S. epidermidis* isolates toward erythromycin, trimethoprim-sulfamethoxazole, penicillin, tetracycline, clindamycin, and gentamicin was 75, 55, 95, 30, 30, and 20%, respectively. Besides, all *S. epidermidis* isolates were susceptible to linezolid. Also, 5, 5, 5, and 15% of isolates were intermediated for erythromycin, tetracycline, clindamycin, and gentamicin antibiotics, respectively. The highest and lowest rates of antibiotic resistance were related to penicillin (95%), and linezolid (0%), respectively. In all, 70% (*n* = 14) of *S. epidermidis* isolates were MDR. Further details of the antibacterial susceptibility pattern of *S. epidermidis* isolates are shown in Table 1.

**TABLE 1:** Antimicrobial susceptibility test in *Staphylococcus epidermidis* isolates.

Isolate (*n* = 20)	Source	FOX	E	TS	P	TE	CD	GM	LZD	MDR/Non-MDR
ATCC 35984 Turkey	−	R	R	R	R	R	R	I	S	MDR
ATCC 35984 Kerman	−	R	R	R	R	S	R	I	S	MDR
DSMZ 3270	−	S	S	S	S	S	S	S	S	Non-MDR
ATCC 12228	−	S	S	S	R	R	S	S	S	Non-MDR
MRSE 1 (Clinical isolate)	Sputum	R	R	R	R	S	R	R	S	MDR
MRSE 2 (Clinical isolate)	Blood	R	R	R	R	S	R	R	S	MDR
MRSE 3 (Clinical isolate)	Blood	R	S	S	R	S	S	S	S	Non-MDR
MSSE 1 (Clinical isolate)	Blood	S	S	S	R	S	S	S	S	Non-MDR
MRSE 4 (Clinical isolate)	Catheter	R	I	S	R	S	S	R	S	Non-MDR
MRSE 5 (Clinical isolate)	Blood	R	R	R	R	S	R	R	S	MDR
MRSE 6 (Clinical isolate)	Blood	R	R	R	R	R	R	S	S	MDR
MRSE 7 (Clinical isolate)	Urine	R	R	R	R	S	S	S	S	MDR
MRSE 8 (Clinical isolate)	Catheter	R	R	R	R	R	S	S	S	MDR
MRSE 9 (Clinical isolate)	Blood	R	R	S	R	S	S	S	S	MDR
MRSE 10 (Clinical isolate)	Urine	R	R	R	R	R	S	S	S	MDR
MRSE 11 (Clinical isolate)	Urine	R	R	S	R	S	S	S	S	MDR
MRSE 12 (Clinical isolate)	Blood	R	R	S	R	R	S	S	S	MDR
MRSE 13 (Clinical isolate)	Wound	R	R	S	R	S	I	S	S	Non-MDR
MRSE 14 (Clinical isolate)	Wound	R	R	R	R	I	S	I	S	MDR
MRSE 15 (Clinical isolate)	Wound	R	R	R	R	S	S	S	S	MDR

*ATCC, American Type Culture Collection; DSMZ, Deutsche Sammlung von Mikroorganismen und Zellkulturen, MRSE, methicillin-resistant S. epidermidis; MSSE, methicillin sensitive S. epidermidis; R, resistant; I, intermediate; S, sensitive; FOX, cefoxitin; E, Erythromycin; TS, Trimethoprim-Sulfamethoxazole; P, Penicillin; TE, Tetracycline; CD, Clindamycin; GM, Gentamicin; LZD, Linezolid; MDR, multidrug-resistant.*

### MIC, MBC, and MBC/MIC Ratio

**FIGURE 1:**
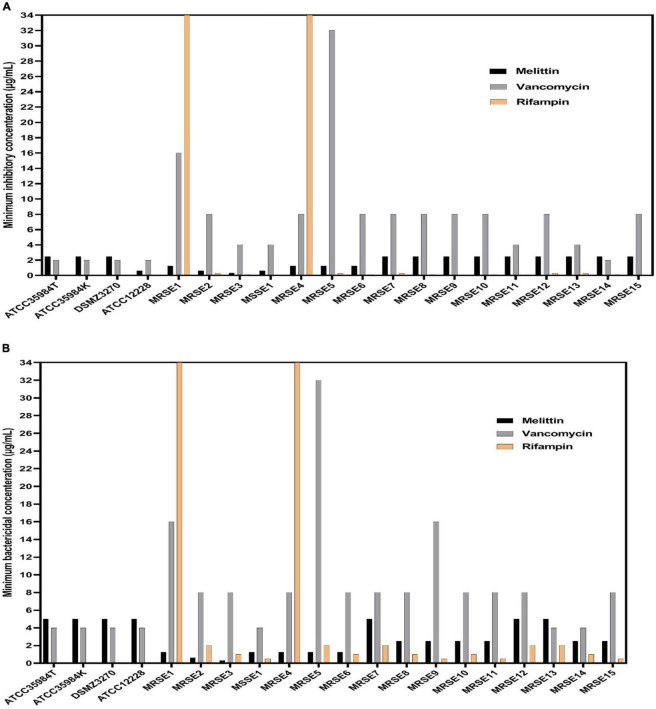
Minimum inhibitory concentration (MIC), minimum bactericidal concentration (MBC) values for vancomycin, rifampin, and melittin against *Staphylococcus epidermidis*. In panel **(A)**, MIC value of rifampin was higher than 1,024 μg/ml for MRSE 1 and MRSE 4 but was not shown due to the space of the graph. In **(B)**, MBC value of rifampin was higher than 1,024 μg/ml for MRSE 1 and MRSE 4 but was not shown due to the space of the graph. ATCC, American Type Culture Collection; DSMZ, Deutsche Sammlung von Mikroorganismen und Zellkulturen, MRSE, methicillin-resistant *S. epidermidis*; MSSE, methicillin sensitive *S. epidermidis*.

**FIGURE 2:**
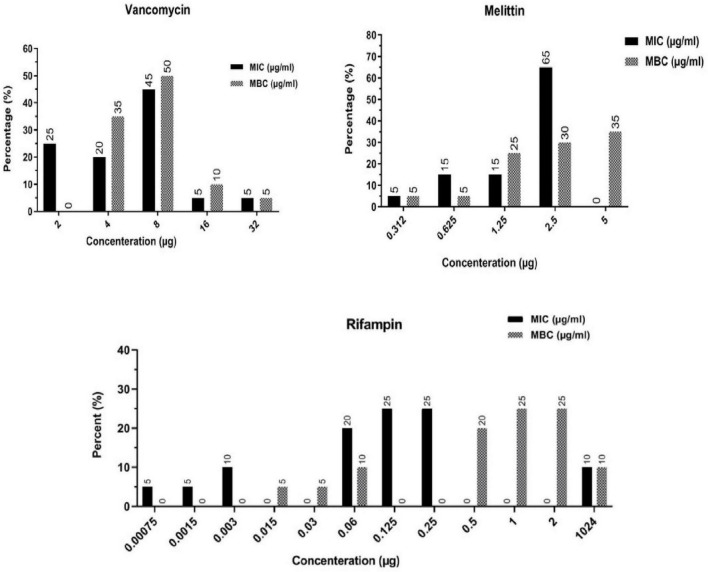
Frequency distribution of MIC and MBC in *Staphylococcus epidermidis* isolates toward vancomycin, rifampin, and melittin. MIC, Minimum inhibitory concentration; MBC, minimum bactericidal concentration.

The findings indicated that melittin inhibited the growth of all *S. epidermidis* isolates, with MIC values ranging from 0.312 to 2.5 μg/ml. Besides, the results also showed the bactericidal activity for melittin against all *S. epidermidis* isolates, with MBC values ranging from 0.312 to 5 μg/ml. The geometric means of MIC values of melittin, vancomycin, and rifampin for *S. epidermidis* isolates were 1.85, 7.3, and 102.5 μg/ml, respectively. The geometric means of the MBC values of melittin, vancomycin, and rifampin for all *S. epidermidis* isolates were 2.85, 8.6, and 103.25 μg/ml, respectively. On the other hand, the MIC_50_ values for melittin, rifampin, and vancomycin were 2.5, 0.125, and 8 μg/ml, respectively, and also, MBC_50_ values for melittin, rifampin, and vancomycin, were 2.5, 1, and 8 μg/ml, respectively. The MIC_90_ values for melittin, rifampin, and vancomycin were 2.5, 0.25, and 16 μg/ml, respectively. And also, MBC_90_ values for melittin, rifampin, and vancomycin, were 5, 2, and 16 μg/ml, respectively. In addition, 45% of isolates were susceptible to vancomycin while 50% of isolates were intermediated (MIC equal to 8–16 μg/ml) to vancomycin, and, unfortunately, we found one vancomycin-resistant MDR-MRSE isolate with MIC equal to 32 μg/ml. Most importantly, our results found rifampin had the MIC range equal to 0.00156- > 1,024 μg/ml. Besides, 10% (*n* = 2) of isolates were resistant to rifampin with MIC higher than 1,024 μg/ml. The geometric means MBC/MIC ratios for vancomycin, rifampin, and melittin were 1.31, 8.41, and 1.41 μg/ml. Further details are displayed in Table 2 and Figures 1, 2.

**TABLE 2:** MBC/MIC ratios for vancomycin, rifampin, and melittin against *Staphylococcus epidermidis.*

Strain	Van-MBC/MIC ratio	Rif-MBC/MIC ratio	Mel-MBC/MIC ratio
ATCC 35984 T	2	16	2
ATCC 35984 K	2	16	2
DSMZ 3270	2	20	2
ATCC 12228	2	2	8
MDR-MRSE 1	1	−	1
MDR-MRSE 2	1	8	1
Non-MDR-MRSE 3	2	8	1
Non-MDR-MSSE 1	1	8	2
Non-MDR-MRSE 4	1	−	1
MDR-MRSE 5	1	8	1
MDR-MRSE 6	1	8	1
MDR-MRSE 7	1	8	2
MDR-MRSE 8	1	8	1
MDR-MRSE 9	2	8	1
MDR-MRSE 10	1	8	1
MDR-MRSE 11	2	8	1
MDR-MRSE 12	1	8	2
Non-MDR-MRSE 13	1	8	2
MDR-MRSE 14	2	8	1
MDR-MRSE 15	1	8	1

*ATCC, American Type Culture Collection; DSMZ, Deutsche Sammlung von Mikroorganismen und Zellkulturen, MRSE, methicillin-resistant S. epidermidis; MSSE, methicillin sensitive S. epidermidis; Van, vancomycin; MIC, minimum inhibitory concentration; MBC, minimum bactericidal concentrations; Rif, rifampin, Mel; melittin; MRSE, methicillin-resistant Staphylococcus epidermidis.*

### Measurement of Synergistic Effects

In the present study, we applied the modified microbroth dilution method to assess the antimicrobial agent interactions by calculating FICi and FBCi values which represent the interaction coefficients. These values indicate whether the combined inhibitory and bactericidal effects of drugs are synergistic, additive, indifferent, and/or antagonistic against selected isolates. In general, combination indexes based on FIC values for melittin–rifampin, and melittin-vancomycin against all isolates were 0.5, and 0.34, respectively. The geometric means of FICi for various melittin–rifampin synergistic concentrations for MDR-MRSE 1, Non-MDR-MRSE 4, ATCC 35984, Non-MDR-MSSE 1, and MDR-MRSE 8 isolates were calculated as 0.24, 0.24, 2, 0.56, and 0.52, respectively (Table 3). The geometric means of FICi for various melittin–vancomycin synergistic concentrations for MDR-MRSE 1, Non-MDR-MSSE 1, MDR-MRSE 8, MDR-MRSE 5, and ATCC 35984 were calculated as 0.32, 0.31, 0.39, 0.3, and 0.42, respectively (Table 4). An unpaired sample *t*-test showed a significant difference between the MIC values of melittin alone and melittin–vancomycin, and melittin–rifampin combinations against MRSE isolates (*p* < 0.0001).

**TABLE 3:** The best synergistic concentrations of Rifampin-Melittin based on MIC against selected isolates.

MRSE 1 and 4^a^		ATCC 35984		MSSE 1		MRSE 8	
Rif + Mel (μ g/ml)	FIC indices	Rif + Mel (μg/ml)	FIC indices	Rif + Mel μg/ml	FIC indices	Rif + Mel (μg/ml)	FIC indices
122^b^ + 0.625	0.61	0.0078 + 1.25	2.5	0.0312^c^ + 0.312	0.74	0.0312^c^ + 1.25	0.74
124 + 0.312	0.37	0.0078 + 0.625	2.25	0.0312 + 0.156	0.62	0.0312 + 0.625	0.49
126 + 0.156	0.24	0.0078 + 0.312	2.12	0.0312 + 0.078	0.55	0.0312 + 0.312	0.37
128 + 0.009	0.13	0.0078 + 0.156	2.06	0.0312 + 0.039	0.53	0.0312 + 0.156	0.31
128 + 0.004	0.12	0.0078 + 0.078	2.02	0.0312 + 0.019	0.51	0.0625 + 0.625	0.75
−	−	0.0078 + 0.039	2.01	0.0312 + 0.009	0.5	0.0625 + 0.312	0.62
−	−	0.0078 + 0.019	2	0.0312 + 0.004	0.502	0.0625 + 0.156	0.56
−	−	0.0078 + 0.009	2	−	−	0.0625 + 0.078	0.52
−	−	0.0078 + 0.004	2	−	−	0.0625 + 0.039	0.51
−	−	0.0039 + 1.25	1.5	−	−	0.0625 + 0.019	0.5
−	−		−	−	−	0.0625 + 0.009	0.5
−	−	−	−	−	−	0.0625 + 0.004	0.5

*FIC, fractional inhibitory concentration; MRSE, methicillin-resistant S. epidermidis; ATCC, American type culture collection; MSSE, methicillin-susceptible S. epidermidis; Rif, rifampin; Mel, melittin; FIC, fractional inhibitory concentration. ^a^These isolates had the same MIC and MBC results, that way, the same synergism formulation was used, and surprisingly, the same results were found again. ^b^The conventional serial dilution for the antibacterial agent interaction assessment has wide intervals for choosing the concentrations needed to enter the antibacterial agents’ interaction, that way, we selected and used novel concentrations other than conventional serial dilutions. ^c^It should be noted various synergism formulations were used for the survey of antibacterial agents’ interaction because of different MIC results for various selected isolates.*

**TABLE 4:** The best synergistic concentrations of Vancomycin-Melittin based on MIC against selected isolates.

MRSE 1		ATCC 35984		MRSE 5		MSSE 1		MRSE 8	
Van + Mel (μ g/ml)	FIC indices	Van + Mel (μg/ml)	FIC indices	Van + Mel (μg/ml)	FIC indices	(Van + Mel) μg/ml	FIC indices	Van + Mel (μg/ml)	FIC indices
8 + 0.312	0.74	1 + 0.625	0.75	16 + 0.312	0.74	2 + 0.156	0.74	4 + 0.625	0.75
8 + 0.156	0.62	1 + 0.312	0.62	16 + 0.156	0.62	1 + 0.312	0.74	4 + 0.312	0.62
8 + 0.078	0.55	1 + 0.156	0.56	16 + 0.078	0.55	1 + 0.156	0.49	4 + 0.156	0.56
6 + 0.039	0.4	1 + 0.078	0.52	16 + 0.039	0.53	0.5 + 0.312	0.62	4 + 0.078	0.52
6 + 0.019	0.39	1 + 0.039	0.51	14 + 0.019	0.45	0.5 + 0.156	0.37	4 + 0.039	0.51
6 + 0.009	0.38	1 + 0.019	0.5	14 + 0.009	0.44	0.5 + 0.078	0.23	4 + 0.019	0.5
6 + 0.004	0.37	1 + 0.009	0.5	14 + 0.004	0.44	0.5 + 0.039	0.18	4 + 0.009	0.5
4 + 0.625	0.75	1 + 0.004	0.5	10 + 0.625	0.81	0.5 + 0.019	0.15	4 + 0.004	0.5
4 + 0.312	0.49	0.5 + 1.25	0.75	10 + 0.312	0.56	0.5 + 0.009	0.13	2 + 1.25	0.75
4 + 0.156	0.37	0.5 + 0.625	0.5	10 + 0.156	0.43	0.5 + 0.004	0.13	2 + 0.625	0.5
4 + 0.078	0.3	0.5 + 0.312	0.37	10 + 0.078	0.36	−	−	2 + 0.312	0.37
4 + 0.039	0.28	0.5 + 0.156	0.31	8 + 0.039	0.28	−	−	2 + 0.156	0.31
4 + 0.019	0.26	0.5 + 0.07	0.27	8 + 0.019	0.26	−	−	2 + 0.078	0.27
4 + 0.009	0.25	0.5 + 0.039	0.26	8 + 0.009	0.25	−	−	2 + 0.039	0.26
4 + 0.004	0.25	0.5 + 0.019	0.25	8 + 0.004	0.25	−	−	2 + 0.019	0.25
2 + 0.625	0.62	0.5 + 0.009	0.25	4 + 0.625	0.62	−	−	2 + 0.009	0.25
2 + 0.312	0.37	0.5 + 0.004	0.25	4 + 0.312	0.37	−	−	2 + 0.004	0.25
2 + 0.156	0.24	−	−	4 + 0.156	0.24	−	−	1 + 1.25	0.62
2 + 0.078	0.18	−	−	4 + 0.078	0.18	−	−	1 + 0.625	0.37
2 + 0.039	0.15	−	−	4 + 0.039	0.15	−	−	1 + 0.312	0.24
2 + 0.019	0.14	−	−	4 + 0.019	0.14	−	−	1 + 0.156	0.18
2 + 0.009	0.13	−	−	4 + 0.009	0.13	−	−	1 + 0.078	0.15
2 + 0.004	0.12	−	−	4 + 0.004	0.12	−	−	0.5 + 1.25	0.56
		−	−	2 + 0.625	0.56	−	−	0.25 + 1.25	0.53
‘		−	−	2 + 0.312	0.26	−	−	−	−
		−	−	2 + 0.156	0.18	−	−	−	−
		−	−	2 + 0.078	0.11	−	−	−	−
		−	−	2 + 0.039	0.09	−	−	−	−

*FIC, fractional inhibitory concentration; MRSE, methicillin-resistant S. epidermidis; ATCC, American type culture collection; MSSE, methicillin-susceptible S. epidermidis; Van, vancomycin; Mel, melittin.*

In particular, based on MIC values, in the two highly rifampin-resistant isolates, MDR-MRSE 1, and Non-MDR-MRSE 4, the maximum synergistic effect with a FICi = 0.12 was found for 128, and 0.0049 μg/ml of rifampin and melittin, respectively, which their MIC values was decreased > 8- and 255.1-fold, respectively. For the MDR-MRSE 8, the maximum synergistic effect with a FICi = 0.31 was found for 0.156, and 0.0312 μg/ml of melittin, and rifampin, respectively, which their MIC values were decreased 4- and 16-fold and, respectively. For the Non-MDR-MSSE 1, the maximum synergistic effect with a FICi = 0.5 was found for 0.0049, and 0.03125 μg/ml of melittin, and rifampin, respectively, which their MIC values were decreased by 2- and 255-fold and, respectively. Interestingly, the *S. epidermidis* ATCC 35984 did not have synergistic effects for all concentration ranges. However, for this strain, the MIC value of the combined form of melittin was decreased 510-fold.

Besides, based on MIC values, for the vancomycin-intermediate MDR-MRSE 1, the maximum synergistic effect with a FICi = 0.12 was found for 0.0049, and 2 μg/ml of melittin, and vancomycin, respectively, which their MIC values were decreased 255- and 8-fold, respectively. For the vancomycin-resistant MDR-MRSE 5, the maximum synergistic effect with a FICi = 0.0.09 was found for 0.039, and 2 μg/ml of melittin, and vancomycin, respectively, which their MIC values were decreased 32- and 16-fold, respectively. For the vancomycin-sensitive *S. epidermidis* ATCC 35984, the maximum synergistic effect with a FICi = 0.18 was found for 0.0049, and 0.5 μg/ml of melittin, and vancomycin, respectively, which their MIC values were decreased 510- and 4-fold, respectively. For the vancomycin-intermediate MDR-MRSE 8, the maximum synergistic effect with a FICi = 0.15 was found for 0.078, and 1 μg/ml of melittin, and vancomycin, respectively, which their MIC values were decreased 32- and 8-fold, respectively. For the vancomycin-sensitive Non-MDR-MSSE, the maximum synergistic effect with a FICi = 0.13 was found for 0.0049, and 0.5 μg/ml of melittin, and vancomycin, respectively, which their MIC values were decreased by 127.5- and 8-fold, respectively.

Most importantly, the interactions of melittin–rifampin and melittin–vancomycin combinations based on MBC values were evaluated at various ranges against selected isolates. In general, combination indexes based on FBC values for melittin–rifampin, and melittin-vancomycin against all isolates were 0.34, and 0.33, respectively. In this regard, the geometric means of FBCi for various melittin–rifampin synergistic concentrations for MDR-MRSE 1, Non-MDR-MSSE 1, MDR-MRSE 8, Non-MDR-MRSE 4, and ATCC 35984 strains were calculated as 0.24, 0.44, 0.3, 0.24, and 0.68, respectively (Table 5). Besides, the geometric means of FBCi for various melittin–vancomycin synergistic concentrations for MDR-MRSE 1, Non-MDR-MSSE 1, MDR-MRSE 8, MDR-MRSE 5, and ATCC 35984 strains were calculated as 0.33, 0.27, 0.42, 0.36, and 0.3, respectively (Table 6). An unpaired sample *t*-test showed a significant difference between MBC values of melittin alone and melittin–vancomycin, and melittin–rifampin combinations against MRSE isolates (*p* = 0.0003).

**TABLE 5:** The best synergistic concentrations of Rifampin-Melittin based on MBC against selected isolates.

MRSE 1, and 4*		ATCC 35984		MSSE 1		MRSE 8	
Rif+Mel (μg/ml)	FBC indices	Rif+Mel (μg/ml)	FBC indices	Rif+Mel (μg/ml)	FBC indices	Rif+Mel (μg/ml)	FBC indices
122+0.625	0.61	0.0312+2.5	0.99	0.25+0.312	0.74	0.5+0.625	0.75
124+0.312	0.37	0.0312+1.25	0.74	0.25+0.156	0.62	0.5+0.312	0.62
126+0.156	0.24	0.0312+0.625	0.62	0.25+0.078	0.55	0.5+0.156	0.56
128+0.009	0.13	0.0312+0.312	0.56	0.25+0.039	0.53	0.5+0.078	0.52
128+0.004	**0.12**	**0.0312+0.156**	**0.53**	0.25+0.019	0.51	0.5+0.039	0.51
−	−	0.0156+2.5	0.74	0.25+0.009	0.5	0.5+0.019	0.5
−	−	−	−	0.25+0.004	0.5	0.5+0.009	0.5
−	−	−	−	0.125+0.625	0.75	0.5+0.004	0.5
−	−	−	−	0.125+0.312	0.49	0.25+1.25	0.75
−	−	−	−	0.125+0.156	0.37	0.25+0.625	0.5
−	−	−	−	0.125+0.078	0.3	0.25+0.312	0.37
−	−	−	−	0.125+0.039	0.28	0.25+0.156	0.31
−	−	−	−	0.0625+0.625	0.625	0.25+0.078	0.27
−	−	−	−	0.0625+0.312	0.37	0.25+0.039	0.26
−	−	−	−	0.0625+0.156	0.24	0.25+0.019	0.25
−	−	−	−	0.0312+0.625	0.56	0.25+0.009	0.25
−	−	−	−	0.0312+0.312	0.31	0.25+0.004	0.25
−	−	−	−	**0.0312+0.156**	**0.18**	0.125+1.25	0.62
−	−	−	−	−	−	0.125+0.625	0.37
−	−	−	−	−	−	0.125+0.312	0.24
−	−	−	−	−	−	0.125+0.156	0.18
−	−	−	−	−	−	0.125+0.078	0.15
−	−	−	−	−	−	0.125+0.039	0.14
−	−	−	−	−	−	0.125+0.019	0.13
−	−	−	−	−	−	0.0625+1.25	0.56
−	−	−	−	−	−	0.0625+0.625	0.31
−	−	−	−	−	−	0.0625+0.312	0.18
−	−	−	−	−	−	0.0625+0.156	0.12
−	−	−	−	−	−	0.0625+0.078	0.09
−	−	−	−	−	−	**0.0625+0.039**	**0.07**
−	−	−	−	−	−	0.0312+1.25	0.53
−	−	−	−	−	−	0.0312+0.625	0.28
−	−	−	−	−	−	0.0312+0.312	0.15

FBC, fractional bactericidal concentration; MRSE, methicillin-resistant S. epidermidis; ATCC, American type culture collection; MSSE, methicillin-susceptible S. epidermidis. *These isolates had the same MBC results, that way, the same synergism formulation was used, and surprisingly, the same results were found again. The best synergistic bactericidal concentrations and their FBC indices for various isolates were bolded.

**TABLE 6:** The best synergistic concentrations of Vancomycin-Melittin based on MBC against selected isolates.

MRSE 1		ATCC 35984		MRSE 5		MSSE 1		MRSE 8	
Van + Mel(μ g/ml)	FBC indices	Van + Mel (μg/ml)	FBC indices	Van + Mel(μg/ml)	FBC indices	Van + Mel μg/ml	FBC indices	Van + Mel (μg/ml)	FBC indices
8 + 0.312	0.74	2 + 1.25	0.75	16 + 0.312	0.74	2 + 0.312	0.74	4 + 0.625	0.75
8 + 0.156	0.62	2 + 0.625	0.62	16 + 0.156	0.62	1 + 0.625	0.75	4 + 0.312	0.62
8 + 0.078	0.55	2 + 0.312	0.56	16 + 0.078	0.55	0.5 + 0.625	0.62	4 + 0.156	0.56
6 + 0.039	0.4	2 + 0.156	0.53	16 + 0.039	0.53	0.5 + 0.312	0.37	4 + 0.078	0.52
6 + 0.019	0.39	2 + 0.078	0.51	14 + 0.019	0.45	0.5 + 0.156	0.24	4 + 0.039	0.51
6 + 0.009	0.38	2 + 0.039	0.5	14 + 0.009	0.44	0.5 + 0.078	0.18	4 + 0.019	0.50
6 + 0.004	0.37	2 + 0.019	0.58	14 + 0.004	0.44	0.5 + 0.039	0.15	4 + 0.009	0.5
4 + 0.625	0.75	2 + 0.009	0.5	10 + 0.625	0.81	0.5 + 0.019	0.14	2 + 1.25	0.75
4 + 0.312	0.49	2 + 0.004	0.5	10 + 0.312	0.56	0.5 + 0.009	0.13	2 + 0.625	0.5
4 + 0.156	0.37	1 + 1.25	0.5	10 + 0.156	0.43	**0.5 + 0.004**	**0.12**	2 + 0.312	0.37
4 + 0.078	0.3	1 + 0.625	0.37	10 + 0.078	0.36	−	−	2 + 0.156	0.31
4 + 0.039	0.28	1 + 0.312	0.312	8 + 0.039	0.28	−	−	2 + 0.078	0.27
4 + 0.019	0.26	1 + 0.156	0.28	8 + 0.019	0.26	−	−	2 + 0.039	0.26
4 + 0.009	0.25	1 + 0.078	0.26	8 + 0.009	0.25	−	−	2 + 0.019	0.25
4 + 0.004	0.25	1 + 0.039	0.25	8 + 0.004	0.25	−	−	1 + 1.25	0.62
2 + 0.625	0.62	1 + 0.019	0.25	4 + 0.625	0.62	−	−	1 + 0.625	0.37
2 + 0.312	0.37	1 + 0.009	0.25	4 + 0.312	0.37	−	−	1 + 0.312	0.24
2 + 0.156	0.24	1 + 0.004	0.25	4 + 0.156	0.24	−	−	**1 + 0.156**	**0.18**
2 + 0.078	0.18	0.5 + 2.5	0.62	4 + 0.078	0.18	−	−	0.5 + 1.25	0.56
2 + 0.039	0.15	0.5 + 1.25	0.37	4 + 0.039	0.15	−	−	−	−
2 + 0.019	0.14	0.5 + 0.625	0.25	**4 + 0.019**	**0.14**	−	−	−	−
**2 + 0.009**	**0.13**	0.5 + 0.312	0.18	2 + 0.625	0.56	−	−	−	−
−	−	0.5 + 0.156	0.15	2 + 0.312	0.26	−	−	−	−
−	−	0.5 + 0.078	0.13	2 + 0.156	0.18	−	−	−	−
−	−	0.5 + 0.039	0.13	−	−	−	−	−	−
−	−	0.5 + 0.019	0.12	−	−	−	−	−	−
−	−	0.5 + 0.009	0.12	−	−	−	−	−	−
−	−	**0.5 + 0.004**	**0.12**	−	−	−	−	−	−
−	−	−	−			−	−	−	−
−	−	−	−	−	−	−	−	−	−

*FBC, fractional bactericidal concentration; MRSE, methicillin-resistant S. epidermidis; ATCC, American type culture collection; MSSE, methicillin-susceptible S. epidermidis. The best synergistic bactericidal concentrations and their FBC indices for various isolates were bolded.*

In particular, for the MDR-MRSE 1, and Non-MDR-MRSE 4, the maximum synergistic effect with an FBCi = 0.12 was found for 0.0049, and 128 μg/ml of melittin, and rifampin, respectively, which their MBC was decreased 255- and > 8-fold, respectively. For the MDR-MRSE 8, the maximum synergistic effect with an FBCi = 0.07 was found for 0.039, and 0.0625 μg/ml of melittin, and rifampin, respectively, which their MBC was decreased 64- and 16-fold, respectively. Besides, an unpaired sample *t*-test showed a significant difference between the FICi and FBCi of melittin–rifampin against MDR-MRSE 8 (*p* = 0.0063). For *S. epidermidis* ATCC 35984, the maximum synergistic effect with an FBCi = 0.53 was found for 0.156, and 0.0312 μg/ml of melittin, and rifampin, respectively, which their MBC was decreased 32- and 2-fold, respectively. Besides, an unpaired sample *t*-test showed a significant difference between the FICi and FBCi of melittin–rifampin against the ATCC 35984 (*p* < 0.0001). For the MSSE 1, the maximum synergistic effect with FBCi = 0.18 was found for 0.156, and 0.0312 μg/ml of melittin, and rifampin, respectively, which their MBC was decreased 8- and 16-fold, respectively.

The interactions of melittin–vancomycin based on MBC values against selected isolates showed that for the MDR-MRSE 1, the maximum synergistic effect with FBCi = 0.13 was found for 0.009, and 2 μg/ml of melittin, and vancomycin, respectively, which their MBC was decreased 138.8- and 8-fold, respectively. For the MDR-MRSE 5, the maximum synergistic effect with FBCi = 0.14 was found for 0.019, and 4 μg/ml for melittin, and vancomycin, respectively, which their MBC was decreased 65.7- and 8-fold, respectively. For the MDR-MRSE 8, the maximum synergistic effect with FBCi = 0.18 was found for 0.156, and 1 μg/ml of melittin, and vancomycin, respectively, which their MBC was decreased 16- and 8-fold, respectively. For the *S. epidermidis* ATCC 35984, the maximum synergistic effect with FBCi = 0.12 was found for 0.0049, and 0.5 μg/ml of melittin, and vancomycin, respectively, which their MBC was decreased 1,020- and 8-fold, respectively. Besides, an unpaired sample *t*-test showed no significant difference between the FICi and FBCi of melittin–vancomycin against the ATCC 35984 (*p* = 0.0668). Finally, for the Non-MDR-MSSE 1, the maximum synergistic effect with FBCi = 0.12 was found for 0.0049, and 0.5 μg/ml of melittin, and vancomycin, respectively, which their MBC was decreased 255- and 8-fold, respectively.

### Serum, and Salt Stability Test for Melittin

To survey the stability of melittin, the antibacterial activity of this peptide against selected strains, and *S. epidermidis* ATCC 35984 was determined. MIC of melittin after the encounter to serum for ATCC 35984, MDR-MRSE 1, Non-MDR-MSSE 1, Non-MDR-MRSE 4, MDR-MRSE 5, and MDR-MRSE 8 were 5, 1.25, 1.25, 2.5, 2.5, and 2.5 μg/ml, respectively. In this regard, a paired sample *t*-test showed no significant difference in the MIC of melittin between standard conditions and serum conditions (*p* > 0.05). Besides, MIC of melittin after the encounter with NaCl, and MgCl_2_ for ATCC 35984, MDR-MRSE 1, Non-MDR-MSSE 1, Non-MDR-MRSE 4, MDR-MRSE 5, and MDR-MRSE 8 were 2.5, 1.25, 0.625, 1.25, 1.25, and 2.5 μg/ml, respectively. In this regard, the sample *t*-test showed no significant difference between the MIC of melittin between standard conditions and NaCl, and MgCl_2_ conditions (*p* > 0.05).

On the other hand, MBC of melittin after serum for ATCC 35984, MDR-MRSE 1, Non-MDR-MSSE 1, Non-MDR-MRSE 4, MDR-MRSE 5, and MDR-MRSE 8 were 10, 2.5, 1.25, 2.5, 1.25, and 5 μg/ml, respectively. In this regard, a paired sample *t*-test showed no significant difference between the MBC of melittin between standard conditions and serum conditions (*p* = 0.0822). Besides, MBC of melittin after the encounter with NaCl, and MgCl_2_ for ATCC 35984, MDR-MRSE 1, Non-MDR-MSSE 1, Non-MDR-MRSE 4, MDR-MRSE 5, and MDR-MRSE 8 were 5, 1.25, 1.25, 1.25, 1.25, and 2.5 μg/ml, respectively. Further details are depicted in Table 7. In this regard, the sample *t*-test showed no significant difference between the MBC of melittin between standard conditions and NaCl, and MgCl_2_ conditions (*p* > 0.05).

**TABLE 7:** Minimum inhibitory concentrations, and minimum bactericidal concentrations of melittin after encounter to serum and salts against selected *Staphylococcus epidermidis strains.*

Strains (*n* = 5)	MIC (μ g/ml)-standard condition	MIC (μ g/ml)-serum	MIC (μ g/ml)- NaCl	MIC (μ g/ml)-MgCl_2_	MBC (μ g/ml)-standard condition	MBC (μ g/ml)-serum	MBC (μ g/ml)-NaCl	MBC (μ g/ml)-MgCl_2_
ATCC 35984	2.5	5	2.5	2.5	5	10	5	5
MDR-MRSE 1	1.25	1.25	1.25	1.25	1.25	2.5	1.25	1.25
Non-MDR-MSSE 1	0.625	1.25	0.625	0.625	1.25	1.25	1.25	1.25
Non-MDR MRSE 4	1.25	2.5	1.25	1.25	1.25	2.5	1.25	1.25
MDR-MRSE 5	1.25	2.5	1.25	1.25	1.25	1.25	1.25	1.25
MDR-MRSE 8	2.5	2.5	2.5	2.5	2.5	5	2.5	2.5

*MIC, minimum inhibitory concentration; MBC, minimum bactericidal concentration; MRSE, methicillin-resistant S. epidermidis; ATCC, American type culture collection; MSSE, methicillin-susceptible S. epidermidis; MDR, multidrug resistance.*

### Cytotoxicity Assays

**FIGURE 3:**
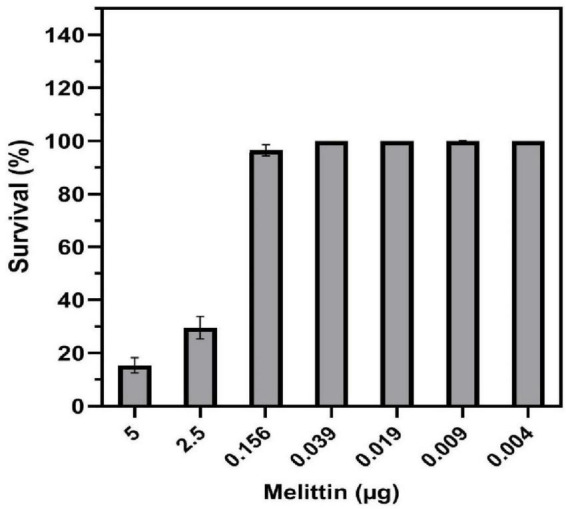
Toxicity of melittin alone and in synergistic concentrations toward human embryonic kidney (HEK) 293 cells.

The cytotoxicity results showed that melittin at concentrations of 5, 2.5, 0.156, 0.039, 0.019, 0.009, and 0.004 μg/ml caused 85.9, 69.4, 3.5, 0, 0, 0, and 0% toxicity on HEK-293 cell, respectively (Figure 3). The paired sample *t*-test showed no significant difference between the survival rate of the synergistic concentrations of melittin and control (*p* = 0.37, and *p* = 0.08, respectively). Notably, the IC50 for melittin was 1.42 μg/ml.

### Hemolytic Activity

**FIGURE 4:**
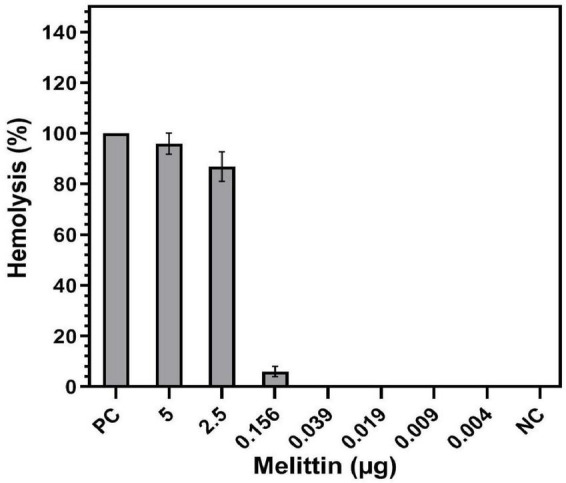
Hemolysis of melittin alone and in synergistic concentrations toward human Red blood cells (RBCs). PC, positive control; NC, negative control.

Hemolytic activity of melittin at the concentration of 5 and 2.5 μg/ml was 91.6 and 80.5%, respectively, whereas melittin at the synergistic concentrations of 0.156, 0.039, 0.019, 0.009, and 0.004 μg/mL showed 6, 0, 0, 0, and 0% hemolysis on human RBCs (Figure 4). Also, the HD50 (a concentration causing 50% hemolysis of RBCs) of melittin was 1.48 μg/ml.

### Determination of Coagulation Effects on Human Plasma

Coagulation effects of melittin in 0.004, 0.009, 0.019, 0.039, 0.156, 2.5, and 5 μg concentrations on extrinsic and intrinsic pathways were evaluated in human plasma by PT and PTT assays, respectively. In this regard, no coagulation activity was seen in PT and PTT assays. Besides, one-way ANOVA showed no significant difference between the PT and PTT of melittin at various doses and control (*p* > 0.05).

### Therapeutic Index of Melittin

The therapeutic index was calculated for melittin alone and at synergistic concentrations with antibiotics. The therapeutic index was 0.57, 7.31, and 18.29 for melittin alone, melittin-rifampin, and melittin-vancomycin, respectively.

## Discussion

Opportunistic infections caused by MRSE strains could be more complicated when they are resistant to other antibacterial drugs (Mirzaei et al., 2019). It has been found that most of these MRSE strains are MDR, and the emergence of MRSE with resistance to vancomycin is particularly of concern (Miragaia et al., 2002; Koch et al., 2020).

In our study, antimicrobial susceptibility patterns of MRSE isolates showed a high level of antibacterial resistance (Table 1). In particular, the antibiotic resistance rates toward erythromycin, trimethoprim-sulfamethoxazole, penicillin, tetracycline, clindamycin, and gentamicin were 75, 55, 95, 30, 30, and 20%, respectively. Among all MRSE isolates, the highest antibiotic resistance of the isolates was against penicillin, followed by erythromycin. The resistance rate to erythromycin was 75%, which is close to previous studies (Kresken et al., 2004; Mirzaei et al., 2019). Similar to a study performed by Wang et al. (2016), our findings indicated that erythromycin and penicillin were the most antibacterial agents that MRSE isolates were resistant to. The resistance rate toward tetracycline was 30%, which is lower than the results of Chabi et al. (Chabi and Momtaz, 2019), and Najar-Peerayeh et al. (2014). Linezolid is highly effective against *S. epidermidis*, however, Linezolid-resistant *S. epidermidis* (LRSE) strains are increasingly observed in some countries (Barros et al., 2014; Bender et al., 2015; Karavasilis et al., 2015; Kosecka-Strojek et al., 2020). We found that all MRSE isolates were susceptible to linezolid, which is consistent with other reports published in Iran (Najar-Peerayeh et al., 2014; Farajzadeh Sheikh et al., 2019). Several studies reported linezolid susceptibility in *Coagulase-negative Staphylococci* (CoNS) and *S. aureus* (De Vecchi et al., 2018; Pereira et al., 2020). However, there are few reports on linezolid resistant *S. aureus* in Iran (Rahimi et al., 2013; Shirvani et al., 2018). Therefore, it should be noted the hazard of linezolid prescription in outpatient cases without obvious indication can increase the potential transmission of its resistance gene into *S. epidermidis* strains. Currently, MDR-MRSE infections have become a major challenge (Miragaia et al., 2007; Ibrahem et al., 2008; Li et al., 2009). In the current study, 70% of isolates were MDR-MRSE which is higher than other reports (Wang et al., 2016; Mirzaei et al., 2019). A previous report published by our team showed that 49.5% of the MRSE isolates were MDR (Mirzaei et al., 2019), and also, Wang et al. (2016) reported 42.6% MDR-MRSE strains in their study.

The high rate of methicillin resistance in Staphylococci has led to the use of antibiotics, such as vancomycin, for the treatment of Staphylococci-associated infections (Mirzaei et al., 2019). However, the resistance to this drug has been reported in CoNS (Schwalbe et al., 1987; Del’alamo et al., 1999). This is a matter of concern because few other options are currently available for the treatment of Staphylococcal infections. In the present study, 50% of isolates showed intermediate resistance (MIC values in a range of 8–16 μg/ml) to vancomycin, and, on top of this, we found one VRSE isolate with a value MIC of 32 μg/ml. Our study is the first report that identified VRSE in Iran. However, strains with decreased susceptibility and resistance to this antibiotic have been found in other countries (Sieradzki et al., 1998; Tacconelli et al., 2001; Nunes et al., 2006). Our results indicated that isolates had an MIC_50_ value of 8 μg/ml against vancomycin. Besides, tolerance to vancomycin has been found in CoNS and *S. aureus* in particular in complicated infections, such as endocarditis, and osteomyelitis especially in immunocompromised patients (Traczewski et al., 2009). In the present study, tolerance to vancomycin was not observed. Most importantly, our results found MIC values in a range between 0.00156 and > 1,024 μg/ml for rifampin, and 10% of rifampin resistance with MIC value higher than 1,024 μg/ml is in agreement with other reports (Najar-Peerayeh et al., 2014). The present report is the first report of MRSE with highly decreased susceptibility to rifampin in Iran. Rifampin is active against Staphylococcal biofilm-associated infections, presumably because of its activity against slow-growing organisms (Wi et al., 2018). However, it has been found that rifampin resistance is easily selected in *Staphylococci* following exposure to low concentrations of rifampin (Wi et al., 2018). In the present study, interestingly, based on the MIC and MBC values of melittin, it was found that all isolates were susceptible to it. The MIC and MBC values of melittin against all isolates were in a range of 0.312–2.5 μg/ml and 0.312–5 μg/ml, respectively. In this line, MIC and MBC values of melittin against rifampin-resistant-MDR-MRSE 1, -Non-MDR-MRSE 4, and vancomycin-resistant-MDR-MRSE 5 were 1.25 μg/ml. Comparing the antibacterial findings of melittin against methicillin-resistant *S. aureus* (MRSA) and *Staphylococcus haemolyticus* in Choi et al. (2015) and Galdiero et al. (2019) indicated a similar finding to our findings.

The overuse of antibiotics in the healthcare systems led to the worldwide spread of antibacterial drug resistance (Sheard et al., 2019). It was previously predicted that annually 10 million people could die from antibacterial drug-resistant associated infections by 2050, however, such prediction can be changed and shortened due to the devastating impact of the Coronavirus disease 2019 (COVID-19) pandemic on the over usage of antibiotics in healthcare systems (Mirzaei et al., 2020). The problem of antimicrobial resistance is especially urgent regarding antibiotic resistance in bacteria (Prestinaci et al., 2015). Regarding this critical situation, AMPs have been described and proposed by Michael Zasloff, Robert E. W. Hancock, and others as a promising approach for the treatment of resistant bacteria (Hancock and Lehrer, 1998; Zasloff, 2002, 2006; Lakshmaiah Narayana and Chen, 2015; Ruden et al., 2019; Zharkova et al., 2019). Furthermore, new resistant strains will inevitably emerge again as the widespread use and misuse of novel drugs with a single target of action (Shang et al., 2019). Combination therapies have been also widely applied toward MDR bacterial infections. It has been demonstrated that AMPs in combination form can allow conventional antibiotics to be more effective toward resistant superbugs (Santos et al., 2021). The combination of AMPs with antibiotics also improves the antibacterial effects of peptides and reduces their toxicity against host cells (Shang et al., 2019). Shang et al. (2019) revealed the synergistic activity of five commercially available antibiotics in combination with AMPs against MDR-MRSE. The synergistic activity of melittin with several antibiotics has been addressed against *Acinetobacter baumannii*, and *Pseudomonas aeruginosa* (Issam et al., 2015; Akbari et al., 2019).

In the present study, the combination of melittin–rifampin, and melittin-vancomycin was evaluated based on MIC values at various ranges against the selected isolates. The geometric means of the FICi for various melittin–rifampin synergistic concentrations against MDR-MRSE 1, Non-MDR-MRSE 4, ATCC 35984, Non-MDR-MSSE 1, and MDR-MRSE 8 were calculated as 0.24, 0.24, 2, 0.56, and 0.52, respectively (Table 3). The geometric means of the FICi for various melittin–vancomycin synergistic concentrations against MDR-MRSE 1, Non-MDR-MSSE 1, MDR-MRSE 8, MDR-MRSE 5, and ATCC 35984 were calculated as 0.32, 0.31, 0.39, 0.3, and 0.42, respectively (Table 4). The combinatorial therapeutic approach can be employed to the enhancement of the inhibitory and bactericidal activity and, more precisely, to prevent the emergence of bacterial resistant mutants in the treatment course (Gopal et al., 2014). In our study, melittin showed a remarkable synergistic effect when combined with vancomycin and rifampin against MDR-MRSE isolates. Pereira et al. (Marques Pereira et al., 2020) reported that the combination of melittin with oxacillin showed synergistic activity with bactericidal effects on MRSA strains. In a pioneer study carried out by Akbari et al. (2019), they showed the highly synergistic effects of melittin with doripenem and ceftazidime antibiotics against MDR-*A. baumannii* and *–P. aeruginosa.* Besides, in a study performed by Zarghami et al. (2021a), the synergistic activity of melittin against MRSA isolates was found when combined with oxacillin and vancomycin. In the present study, it is worth mentioning that the MIC values of vancomycin and rifampin in combination with melittin in some combined concentrations were not altered, resulting in indifferent and/or antagonism interactions between melittin and the examined antimicrobial agents.

Most importantly, we evaluated the interaction between antimicrobial agents based on their MBC values, and hence, FBCi was determined. This index is very critical for analyzing the combination of antibacterial agents because the concentration for eradication of drug-resistant bacteria is MBC concentration and not MIC, but this point has been ignored in all studies performed to date. The geometric means of the FBCi for various melittin–rifampin concentrations against MDR-MRSE 1, Non-MDR-MRSE 4, Non-MDR-MSSE 1, MDR-MRSE 8, and ATCC 35984 were calculated as 0.24, 0.44, 0.3, 0.24, and 0.68, respectively (Table 5). Besides, the geometric means of the FBCi for various melittin–vancomycin concentrations against MDR-MRSE 1, Non-MDR-MSSE 1, MDR-MRSE 8, MDR-MRSE 5, and ATCC 35984 were calculated as 0.33, 0.27, 0.42, 0.36, and 0.3, respectively (Table 6). An unpaired sample *t*-test showed a significant difference between the MBC of melittin alone and in combination with antibiotics against MRSE isolates (*p* < 0.0001).

By means of the determination of FICi and FBCi for antimicrobial agents, the precise information about their bacteriostatic and bactericidal effects can be deduced in combination. In the present study, the difference between the bactericidal and inhibitory properties of antimicrobial agents in the synergistic form was smaller than in the single form. For bactericidal agents whose MIC and MBC are equal, the FIC indices can be predictive of FBC indices and bactericidal synergy. As mentioned above, the MBC is the concentration for the killing of bacteria, and FBC based on MBC should be determined, so we suggest researchers determine FICi and FBCi simultaneously when evaluating the combination of antibacterial agents.

In this regard, a study by Mackay et al. (2000) found that synergy by FIC index predicted synergy by FBC index at only 67% of antibiotic-combination assays. In summary, they found a good reproducibility of the chequerboard technique and a good correlation between FIC and FBC indices when all tests are performed simultaneously in the same system.

Besides, our results also showed that interactions of melittin–rifampin combinations are strain-dependent and the maximum of synergism was found in rifampin-resistant strains whilst rifampin susceptible strains synergism was weakly found and, even, in very susceptible-strain, antagonism was found. One of our results of this study was addressed the following question: Can synergism and antagonism be found with a given pair of antibacterial agents depending upon the relative proportion of them in the mixture? In this regard, the quantitative data of the present work answered this question. Our results also showed that the interaction of melittin–rifampin combinations is concentration-dependent, which for providing highly synergism effects in combined antimicrobial agents, the best formulation of them was needed. Our results showed that when the ranges of concentrations of pairs of antibacterial agents are studied, it is possible that just a narrow range can show synergistic in their action. Besides, in resistant bacterial isolates, the additive, and synergistic concentration ranges are wider than the sensitive isolates (Gunnison et al., 1953). In conclusion, this study presented a modified chequerboard technique and confirmed a significant correlation between FIC and FBC indices for bactericidal agents whilst it is not good predictive when one of the pairs is a bacteriostatic agent. Taken together, our results are interesting in particular for *in vivo* research because the higher doses of antimicrobial agents must be administrated to eradicate the infection that prevents the recurrence although they increase the side effects on the host. Finally, these findings can change the required dosages for clinical trials and be helpful in therapies.

It has been noted that melittin binds to bacterial membranes and produces pores, resulting in osmotic bacterial lysis (Bevalian et al., 2021). Interestingly, the induced synergism of melittin with vancomycin and rifampin is most likely related to their site of action on the bacterial membrane, cell wall, and RNA polymerase. Melittin disrupts the integrity of the cell membrane and creates pores that likely facilitate the penetration of rifampin and vancomycin inside the bacteria, and at the next step, rifampin causes the inhibition of bacterial growth through the inhibition of RNA polymerase (Lee et al., 2013; Światły-Błaszkiewicz et al., 2020). And also vancomycin causes death in bacteria *via* the inhibition of the synthesis of the bacterial cell wall (Zarghami et al., 2021a). These data are in agreement with the results of Zarghami et al. (2021a) which showed synergism between melittin and vancomycin.

In the present study, although the antibacterial activity of melittin against *S. epidermidis* strains was reduced in MHB containing human serum, it was still completely active to inhibit and kill the bacterial cells at low doses (Table 7). In this regard, no significant difference was found between MIC, and MBC doses of melittin in MHB and MHB containing 10% human serum (*p* = 0.0599, and *p* = 0.0822, respectively). It should be noted that anionic proteins, such as albumin, can bind to melittin, reducing their availability, and/or to the bacterial surface, and masking the peptide-binding sites may be one reason for the reduction of antibacterial activity as previously found for other AMPs like PV3, and human beta-defensin-3 (HbetaD-3) (Maisetta et al., 2008; Memariani et al., 2016). Besides, in the present study, in the presence of salt or serum, the MIC and MBC doses of melittin against all *S. epidermidis* strains ranged from 0.625 to 10 μg/ml. Compared with the standard condition, there was not seen any decrease in antibacterial activity, including the MIC, and MBC of melittin against strains under 150 mM NaCl and 1 mM MgCl2 (*p* > 0.99), showing that melittin has full stability in MHB containing salts. Our result is in accordance with Memariani et al. (2016) that found peptide PV3 does not have significant differences between MIC, and MBCs of PV3 in MHB and MHB containing 10% human serum, 150 mM NaCl, and 1 mM MgCl_2_. Conversely, some studies reported that salts can reduce the activity of some AMPs (Uccelletti et al., 2010; Xu et al., 2014).

Apart from the prevention of the emergence of drug resistance mutant, another aim of combination therapy is to achieve greater efficacy utilizing lower-dose combinations compared with higher-dose monotherapy approaches, and in this regard, various studies of greater efficacy displayed by compound combinations toward monotherapy have been performed (Zimmermann et al., 2007; Ejim et al., 2011; Tamma et al., 2012). This can potentially cause a lower risk of side effects of antibacterial agents and, a better quality of life for patients (Patel and Saravolatz, 2006). In this study, to provide insight into potential cell toxicity, we tested the cytotoxicity of melittin at synergism concentrations using the MTT assay on the HEK-293 cell line. The results showed that synergistic concentrations of melittin were needed to kill bacterial isolates no considerable cytotoxicity was observed after 24 h, whilst this peptide showed 85.9% toxicity when used alone (Table 7 and Figure 2). On the other hand, the reduction of the dosage of vancomycin and rifampin in combination with melittin can decrease their side effects particularly in patients with renal impairment. Increased drug clearance results in lower drug concentrations, while decreased drug clearance leads to higher drug concentrations and thus higher drug efficacy. To avoid tissue injury, when drug clearance is significantly decreased, the dose of renally cleared drugs should usually be diminished in patients with renal disease. According to our results, induced synergism between melittin and antibiotics led to a decrease in the required concentrations of melittin, rifampin, and vancomycin by 8–1,020-fold, 2–16-fold, and 4–16-fold, respectively. These results are in accordance with the cytotoxicity results reported by Akbari et al. (2019). This phenomenon represents that the combination of melittin with vancomycin and rifampin could be a potential candidate for the treatment of infections caused by MDR-MRSE.

It has been noted that melittin can intercalate into the membrane of human RBCs (Światły-Błaszkiewicz et al., 2020). Many studies have found that melittin is involved in the disruption of phospholipids packaging in the lipid bilayer, pore formation, membrane protein aggregation, and lysis (Światły-Błaszkiewicz et al., 2020). The present study provided insight into the hemolytic activity of melittin alone and in synergistic concentrations on human RBCs. Melittin did not show hemolytic activity in synergistic concentration, whereas this peptide showed 96% hemolytic activity when used alone (Figure 3). These findings are in accordance with the results obtained by Zarghami et al. (2021a) who found that melittin had no hemolytic effect at the low concentrations. In the present study, no coagulation activity was found for synergistic and alone concentrations of melittin in both PTT and PT assays (Figure 4), and ANOVA showed no significant difference between the PT and PTT of melittin at various melittin concentrations and control (0 > 0.05). These results are in accordance with previous reports that peptides, such as LL-37 (the C-terminal peptide of human cathelicidin AMP) and Polymyxin B, do not affect the plasmatic coagulation of heparinized and citrate containing plasma [74]. Finally, our findings showed that the therapeutic index of melittin was improved 32.08- and 12.82-fold when combined with vancomycin and rifampin, respectively. The highest therapeutic index was found for melittin when combined with vancomycin. The therapeutic index could be increased in one of the following three ways: increasing antimicrobial activity, decreasing hemolytic activity while maintaining antimicrobial activity, or a combination of both increasing antimicrobial activity and decreasing hemolytic activity (Chen et al., 2005).

## Conclusion

Based on our findings, melittin was effective against MDR-MRSE isolates and this AMP also showed highly synergistic effects when combined with vancomycin and rifampin. This evidence provides hope for the treatment of the challenging rifampin-resistant, vancomycin-intermediate, and vancomycin-resistant MDR-MRSE isolates. The data also showed that the synergism of melittin-vancomycin was higher than that of melittin–rifampin. Furthermore, the highly induced synergism between melittin, vancomycin, and rifampin led to a decrease in their concentration so that the effective melittin concentrations needed to kill bacteria had no cytotoxicity, hemolytic, and coagulation activities. Accordingly, the therapeutic index of melittin was improved 32.08- and 12.82-fold when combined with vancomycin and rifampin, respectively. Interestingly, the antibacterial activity of melittin did not reduce encounter human serum. In summary, both combinations of melittin–vancomycin, and melittin–rifampin could be a useful strategy for the treatment of MDR-MRSE infections. The potential advantages of antimicrobial combination therapy include increased efficacy, prevention of the emergence of resistant strains, increased bactericidal activity, reduced side effects as well as a reduction in costs of treatment.

## Data Availability Statement

The raw data supporting the conclusion of this article will be available at the direct request of the corresponding authors.

## Author Contributions

RM performed all experiments and analyses, wrote the manuscript, and contributed to the supervisor. MA, CA, IS, GI, and EJ served as advisors. KB suggested the implementation of melittin, contributed to the supervisor, and writing, revision, and redaction of the manuscript. All authors contributed to the article and approved the submitted version.

## Conflict of Interest

The authors declare that the research was conducted in the absence of any commercial or financial relationships that could be construed as a potential conflict of interest.

## Publisher’s Note

All claims expressed in this article are solely those of the authors and do not necessarily represent those of their affiliated organizations, or those of the publisher, the editors and the reviewers. Any product that may be evaluated in this article, or claim that may be made by its manufacturer, is not guaranteed or endorsed by the publisher.

## References

[B1] Aghazadeh H. Memariani H. Ranjbar R. Pooshang Bagheri K. (2019). The activity and action mechanism of novel short selective LL-37-derived anticancer peptides against clinical isolates of *Escherichia coli*. *Chem. Biol. Drug Des.* 93 75–83. 30120878 10.1111/cbdd.13381

[B2] Akbari R. Hakemi-Vala M. Pashaie F. Bevalian P. Hashemi A. Pooshang Bagheri K. (2019). Highly synergistic effects of melittin with conventional antibiotics against multidrug-resistant isolates of *Acinetobacter baumannii* and *Pseudomonas aeruginosa*. *Microb. Drug Resist.* 25 193–202. 30281385 10.1089/mdr.2018.0016

[B3] Arciola C. R. Baldassarri L. Montanaro L. (2002). In catheter infections by *Staphylococcus epidermidis* the intercellular adhesion (ica) locus is a molecular marker of the virulent slime−producing strains. *J. Biomed. Mater. Res.* 59 557–562. 10.1002/jbm.10006 11774314

[B4] Arciola C. R. Campoccia D. Baldassarri L. Donati M. E. Pirini V. Gamberini S. (2006). Detection of biofilm formation in *Staphylococcus epidermidis* from implant infections. Comparison of a PCR-method that recognizes the presence of ica genes with two classic phenotypic methods. *J. Biomed. Mater. Res. A* 76 425–430. 10.1002/jbm.a.30552 16270350

[B5] Barceló-Vidal J. Rodríguez-García E. Grau S. (2018). Extremely high levels of vancomycin can cause severe renal toxicity. *Infect. Drug Resist.* 11 1027–1030. 10.2147/IDR.S171669 30104890 PMC6071627

[B6] Bardbari A. M. Arabestani M. R. Karami M. Keramat F. Aghazadeh H. Alikhani M. Y. (2018). Highly synergistic activity of melittin with imipenem and colistin in biofilm inhibition against multidrug-resistant strong biofilm producer strains of *Acinetobacter baumannii*. *Eur. J. Clin. Microbiol. Infect. Dis.* 37 443–454. 10.1007/s10096-018-3189-7 29353377

[B7] Barros M. Branquinho R. Grosso F. Peixe L. Novais C. (2014). Linezolid-resistant *Staphylococcus epidermidis*, Portugal, 2012. *Emerg. Infect. Dis.* 20 903–905. 10.3201/eid2005.130783 24751182 PMC4012793

[B8] Bender J. Strommenger B. Steglich M. Zimmermann O. Fenner I. Lensing C. (2015). Linezolid resistance in clinical isolates of *Staphylococcus epidermidis* from German hospitals and characterization of two cfr-carrying plasmids. *J. Antimicrob. Chemother.* 70 1630–1638. 10.1093/jac/dkv025 25740949

[B9] Bevalian P. Pashaei F. Akbari R. Bagheri K. P. (2021). Eradication of vancomycin-resistant *Staphylococcus aureus* on a mouse model of third-degree burn infection by melittin: an antimicrobial peptide from bee venom. *Toxicon* 199 49–59. 10.1016/j.toxicon.2021.05.015 34087287

[B10] Bloemendaal A. L. Brouwer E. C. Fluit A. C. (2010). Methicillin resistance transfer from *Staphylocccus epidermidis* to methicillin-susceptible *Staphylococcus aureus* in a patient during antibiotic therapy. *PLoS One* 5:e11841. 10.1371/journal.pone.0011841 20686601 PMC2912275

[B11] Chabi R. Momtaz H. (2019). Virulence factors and antibiotic resistance properties of the *Staphylococcus epidermidis* strains isolated from hospital infections in Ahvaz, Iran. *Trop. Med. Health* 47:56. 10.1186/s41182-019-0180-7 31844416 PMC6896349

[B12] Chen Y. Mant C. T. Farmer S. W. Hancock R. E. Vasil M. L. Hodges R. S. (2005). Rational design of α-helical antimicrobial peptides with enhanced activities and specificity/therapeutic index. *J. Biol. Chem.* 280 12316–12329. 10.1074/jbc.M413406200 15677462 PMC1393284

[B13] Cherifi S. Byl B. Deplano A. Nonhoff C. Denis O. Hallin M. (2013). Comparative epidemiology of *Staphylococcus epidermidis* isolates from patients with catheter-related bacteremia and from healthy volunteers. *J. Clin. Microbiol.* 51 1541–1547. 10.1128/JCM.03378-12 23486718 PMC3647944

[B14] Cherubin C. E. Corrado M. L. Sierra M. F. Gombert M. E. Shulman M. (1981). Susceptibility of gram-positive cocci to various antibiotics, including cefotaxime, moxalactam, and N-formimidoyl thienamycin. *Antimicrob. Agents Chemother.* 20 553–555. 10.1128/AAC.20.4.553 6282200 PMC181744

[B15] Choi J. H. Jang A. Y. Lin S. Lim S. Kim D. Park K. (2015). Melittin, a honeybee venom-derived antimicrobial peptide, may target methicillin-resistant *Staphylococcus aureus*. *Mol. Med. Rep.* 12 6483–6490. 10.3892/mmr.2015.4275 26330195 PMC4626175

[B16] De Vecchi E. George D. A. Romanò C. L. Pregliasco F. E. Mattina R. Drago L. (2018). Antibiotic sensitivities of coagulase-negative staphylococci and *Staphylococcus aureus* in hip and knee periprosthetic joint infections: does this differ if patients meet the International Consensus Meeting Criteria? *Infect. Drug Resist.* 11 539–546. 10.2147/IDR.S151271 29695923 PMC5905490

[B17] Del’alamo L. Cereda R. F. Tosin I. Miranda E. A. Sader H. S. (1999). Antimicrobial susceptibility of coagulase-negative staphylococci and characterization of isolates with reduced susceptibility to glycopeptides. *Diagn. Microbiol. Infect. Dis.* 34 185–191. 10.1016/s0732-8893(99)00034-6 10403098

[B18] Dezfuli H. T. Shahbazzadeh D. Eidi A. Bagheri K. P. Pakravan N. Amini S. (2014). Induction of IFN-γ cytokine response against hepatitis B surface antigen using melittin. *Gastroenterol. Hepatol. Bed Bench* 7 108–117. 24834302 PMC4017562

[B19] Eisapoor S. S. Jamili S. Shahbazzadeh D. Ghavam Mostafavi P. Pooshang Bagheri K. (2016). A new, high yield, rapid, and cost-effective protocol to deprotection of cysteine-rich conopeptide, omega-conotoxin MVIIA. *Chem. Biol. Drug Des.* 87 687–693. 10.1111/cbdd.12702 26662374

[B20] Ejim L. Farha M. A. Falconer S. B. Wildenhain J. Coombes B. K. Tyers M. (2011). Combinations of antibiotics and nonantibiotic drugs enhance antimicrobial efficacy. *Nat. Chem. Biol.* 7 348–350. 10.1038/nchembio.559 21516114

[B21] Eom J. S. Kim W. J. (2001). Antibiotic treatment of methicillin-resistant *Staphylococcus epidermidis* (MRSE) infection. *J. Korean Med. Assoc.* 44 1232–1240.

[B22] Farajzadeh Sheikh A. Asareh Zadegan Dezfuli A. Navidifar T. Fard S. S. Dehdashtian M. (2019). Association between biofilm formation, structure and antibiotic resistance in *Staphylococcus epidermidis* isolated from neonatal septicemia in southwest Iran. *Infect. Drug Resist.* 12 1771–1782. 10.2147/IDR.S204432 31303772 PMC6603288

[B23] Galdiero E. Siciliano A. Gesuele R. Di Onofrio V. Falanga A. Maione A. (2019). Melittin inhibition and eradication activity for resistant polymicrobial biofilm isolated from a dairy industry after disinfection. *Int. J. Microbiol.* 2019:4012394. 10.1155/2019/4012394 30766602 PMC6350607

[B24] Gidari A. Sabbatini S. Schiaroli E. Perito S. Francisci D. Baldelli F. (2020). Tedizolid-rifampicin combination prevents rifampicin-resistance on in vitro model of *Staphylococcus aureus* mature biofilm. *Front. Microbiol.* 11:2085. 10.3389/fmicb.2020.02085 32983061 PMC7484889

[B25] Gómez-Sanz E. Ceballos S. Ruiz-Ripa L. Zarazaga M. Torres C. (2019). Clonally diverse methicillin and multidrug resistant coagulase negative staphylococci are ubiquitous and pose transfer ability between pets and their owners. *Front. Microbiol.* 10:485. 10.3389/fmicb.2019.00485 30972035 PMC6443710

[B26] Gopal R. Kim Y. G. Lee J. H. Lee S. K. Chae J. D. Son B. K. (2014). Synergistic effects and antibiofilm properties of chimeric peptides against multidrug-resistant *Acinetobacter baumannii* strains. *Antimicrob. Agents Chemother.* 58 1622–1629. 10.1128/AAC.02473-13 24366740 PMC3957903

[B27] Gunnison J. Shevky M. Bruff J. Coleman V. Jawetz E. (1953). Studies on antibiotic synergism and antagonism: the effect in vitro of combinations of antibiotics on bacteria of varying resistance to single antibiotics. *J. Bacteriol.* 66 150–158. 10.1128/jb.66.2.150-158.1953 13084551 PMC357114

[B28] Hancock R. E. Lehrer R. (1998). Cationic peptides: a new source of antibiotics. *Trends Biotechnol.* 16 82–88. 10.1016/s0167-7799(97)01156-6 9487736

[B29] Horan T. C. Andrus M. Dudeck M. A. (2008). CDC/NHSN surveillance definition of health care–associated infection and criteria for specific types of infections in the acute care setting. *Am. J. Infect. Control* 36 309–332. 10.1016/j.ajic.2008.03.002 18538699

[B30] Howden B. P. Davies J. K. Johnson P. D. Stinear T. P. Grayson M. L. (2010). Reduced vancomycin susceptibility in *Staphylococcus aureus*, including vancomycin-intermediate and heterogeneous vancomycin-intermediate strains: resistance mechanisms, laboratory detection, and clinical implications. *Clin. Microbiol. Rev.* 23 99–139. 10.1128/CMR.00042-09 20065327 PMC2806658

[B31] Ibrahem S. Salmenlinna S. Lyytikäinen O. Vaara M. Vuopio-Varkila J. (2008). Molecular characterization of methicillin-resistant *Staphylococcus epidermidis* strains from bacteraemic patients. *Clin. Microbiol. Infect.* 14 1020–1027. 10.1111/j.1469-0691.2008.02080.x 19040473

[B32] Issam A.-A. Zimmermann S. Reichling J. Wink M. (2015). Pharmacological synergism of bee venom and melittin with antibiotics and plant secondary metabolites against multi-drug resistant microbial pathogens. *Phytomedicine* 22 245–255. 10.1016/j.phymed.2014.11.019 25765829

[B33] Jamasbi E. Mularski A. Separovic F. (2016). Model membrane and cell studies of antimicrobial activity of melittin analogues. *Curr. Top. Med. Chem.* 16 40–45. 10.2174/1568026615666150703115919 26139117

[B34] Ju O. Woolley M. Gordon D. (2006). Emergence and spread of rifampicin-resistant, methicillin-resistant *Staphylococcus aureus* during vancomycin–rifampicin combination therapy in an intensive care unit. *Eur. J. Clin. Microbiol. Infect. Dis.* 25 61–62. 10.1007/s10096-005-0063-1 16331332

[B35] Karaaslan E. Dosler S. Gerceker A. A. (2016). Antibacterial and anti-biofilm activities of melittin and colistin, alone and in combination with antibiotics against Gram-negative bacteria. *J. Chemother.* 28 95–103. 10.1179/1973947815Y.0000000004 25801062

[B36] Karavasilis V. Zarkotou O. Panopoulou M. Kachrimanidou M. Themeli-Digalaki K. Stylianakis A. (2015). Wide dissemination of linezolid-resistant *Staphylococcus epidermidis* in Greece is associated with a linezolid-dependent ST22 clone. *J. Antimicrob. Chemother.* 70 1625–1629. 10.1093/jac/dkv028 25712317

[B37] Khozani R. S. Shahbazzadeh D. Harzandi N. Feizabadi M. M. Bagheri K. P. (2019). Kinetics study of antimicrobial peptide, melittin, in simultaneous biofilm degradation and eradication of potent biofilm producing MDR *Pseudomonas aeruginosa* isolates. *Int. J. Pept. Res. Ther.* 25 329–338.

[B38] Koch J. A. Pust T. M. Cappellini A. J. Mandell J. B. Ma D. Shah N. B. (2020). *Staphylococcus epidermidis* biofilms have a high tolerance to antibiotics in periprosthetic joint infection. *Life* 10:253. 10.3390/life10110253 33114423 PMC7693748

[B39] Kosecka-Strojek M. Sadowy E. Gawryszewska I. Klepacka J. Tomasik T. Michalik M. (2020). Emergence of linezolid-resistant *Staphylococcus epidermidis* in the tertiary children’s hospital in Cracow, Poland. *Eur. J. Clin. Microbiol. Infect. Dis.* 39 1717–1725. 10.1007/s10096-020-03893-w 32350737 PMC7427702

[B40] Krcmery V. Trupl J. Drgona L. Lacka J. Kukuckova E. Oravcova E. (1996). Nosocomial bacteremia due to vancomycin-resistant *Staphylococcus epidermidis* in four patients with cancer, neutropenia, and previous treatment with vancomycin. *Eur. J. Clin. Microbiol. Infect. Dis.* 15 259–261. 10.1007/BF01591369 8740867

[B41] Kresken M. Hafner D. Schmitz F.-J. Wichelhaus T. A. (2004). Prevalence of mupirocin resistance in clinical isolates of *Staphylococcus aureus* and *Staphylococcus epidermidis*: results of the antimicrobial resistance surveillance study of the Paul-Ehrlich-society for chemotherapy, 2001. *Int. J. Antimicrob. Agents* 23 577–581. 10.1016/j.ijantimicag.2003.11.007 15194128

[B42] Lakshmaiah Narayana J. Chen J. Y. (2015). Antimicrobial peptides: possible anti-infective agents. *Peptides* 72 88–94. 10.1016/j.peptides.2015.05.012 26048089

[B43] Lee M. T. Sun T. L. Hung W. C. Huang H. W. (2013). Process of inducing pores in membranes by melittin. *Proc. Natl. Acad. Sci. U.S.A.* 110 14243–14248. 10.1073/pnas.1307010110 23940362 PMC3761581

[B44] Li M. Wang X. Gao Q. Lu Y. (2009). Molecular characterization of *Staphylococcus epidermidis* strains isolated from a teaching hospital in Shanghai, China. *J. Med. Microbiol.* 58 456–461. 10.1099/jmm.0.007567-0 19273641

[B45] Mackay M. Milne K. Gould I. (2000). Comparison of methods for assessing synergic antibiotic interactions. *Int. J. Antimicrob. Agents* 15 125–129. 10.1016/s0924-8579(00)00149-7 10854808

[B46] Magiorakos A.-P. Srinivasan A. Carey R. T. Carmeli Y. Falagas M. T. Giske C. T. (2012). Multidrug-resistant, extensively drug-resistant and pandrug-resistant bacteria: an international expert proposal for interim standard definitions for acquired resistance. *Clin. Microbiol. Infect.* 18 268–281. 10.1111/j.1469-0691.2011.03570.x 21793988

[B47] Maisetta G. Di Luca M. Esin S. Florio W. Brancatisano F. L. Bottai D. (2008). Evaluation of the inhibitory effects of human serum components on bactericidal activity of human beta defensin 3. *Peptides* 29 1–6. 10.1016/j.peptides.2007.10.013 18045738

[B48] Marques Pereira A. F. Albano M. Bergamo Alves F. C. Murbach Teles Andrade B. F. Furlanetto A. Mores Rall V. L. (2020). Influence of apitoxin and melittin from *Apis mellifera* bee on *Staphylococcus aureus* strains. *Microb. Pathog.* 141:104011. 10.1016/j.micpath.2020.104011 32004624

[B49] Memariani H. Memariani M. Shahidi-Dadras M. Nasiri S. Akhavan M. M. Moravvej H. (2019). Melittin: from honeybees to superbugs. *Appl. Microbiol. Biotechnol.* 103 3265–3276. 10.1007/s00253-019-09698-y 30824944

[B50] Memariani H. Shahbazzadeh D. Sabatier J.-M. Memariani M. Karbalaeimahdi A. Bagheri K. P. (2016). Mechanism of action and in vitro activity of short hybrid antimicrobial peptide PV3 against *Pseudomonas aeruginosa*. *Biochem. Biophys. Res. Commun.* 479 103–108. 10.1016/j.bbrc.2016.09.045 27622323

[B51] Memariani H. Shahbazzadeh D. Sabatier J. M. Pooshang Bagheri K. (2018). Membrane-active peptide PV 3 efficiently eradicates multidrug-resistant *Pseudomonas aeruginosa* in a mouse model of burn infection. *Apmis* 126 114–122. 10.1111/apm.12791 29327480

[B52] Miragaia M. Couto I. Pereira S. F. Kristinsson K. G. Westh H. Jarløv J. O. (2002). Molecular characterization of methicillin-resistant *Staphylococcus epidermidis* clones: evidence of geographic dissemination. *J. Clin. Microbiol.* 40 430–438. 10.1128/JCM.40.2.430-438.2002 11825953 PMC153385

[B53] Miragaia M. Thomas J. C. Couto I. Enright M. C. De Lencastre H. (2007). Inferring a population structure for *Staphylococcus epidermidis* from multilocus sequence typing data. *J. Bacteriol.* 189 2540–2552. 10.1128/JB.01484-06 17220222 PMC1899367

[B54] Mirzaei R. Alikhani M. Y. Arciola C. R. Sedighi I. Yousefimashouf R. Bagheri K. P. (2022). Prevention, inhibition, and degradation effects of melittin alone and in combination with vancomycin and rifampin against strong biofilm producer strains of methicillin-resistant *Staphylococcus epidermidis*. *Biomed. Pharmacother.* 147:112670. 10.1016/j.biopha.2022.112670 35123230

[B55] Mirzaei R. Goodarzi P. Asadi M. Soltani A. Aljanabi H. A. A. Jeda A. S. (2020). Bacterial co-infections with SARS-CoV-2. *IUBMB Life* 72 2097–2111. 32770825 10.1002/iub.2356 PMC7436231

[B56] Mirzaei R. Kalani B. S. Moghadam M. S. Mahdiun F. Irajian G. (2019). Multilocus variable number tandem repeat analysis and antimicrobial susceptibility pattern of *Staphylococcus epidermidis* isolates in Tehran, Iran. *Rev. Med. Microbiol.* 30 95–108.

[B57] Murray P. R. Baron E. Jorgensen J. Pfaller M. Yolken R. (2009). *Manual of Clinical Microbiology 2007.* Washington, DC: American Society for Microbiology.

[B58] Najar-Peerayeh S. Moghadas A. J. Behmanesh M. (2014). Antibiotic susceptibility and mecA frequency in *Staphylococcus epidermidis*, isolated from intensive care unit patients. *Jundishapur J. Microbiol.* 7:e11188. 10.5812/jjm.11188 25485050 PMC4255212

[B59] Nunes A. P. F. Teixeira L. M. Iorio N. L. P. Bastos C. C. R. De Sousa Fonseca L. Souto-Padrón T. (2006). Heterogeneous resistance to vancomycin in *Staphylococcus epidermidis*, *Staphylococcus haemolyticus* and *Staphylococcus warneri* clinical strains: characterisation of glycopeptide susceptibility profiles and cell wall thickening. *Int. J. Antimicrob. Agents* 27 307–315. 10.1016/j.ijantimicag.2005.11.013 16542825

[B60] Ong C. T. Nicolau D. P. (2004). “Chapter 8 − Rationale and utility of therapeutic drug monitoring for the optimization of antibiotic therapy,” in *Handbook of Analytical Separations*, ed. Hempel G. (Amsterdam: Elsevier Science B.V), 195–219.

[B61] Otto M. (2009). *Staphylococcus epidermidis*—the ‘accidental’ pathogen. *Nat. Rev. Microbiol.* 7 555–567. 10.1038/nrmicro2182 19609257 PMC2807625

[B62] Pashaei F. Bevalian P. Akbari R. Bagheri K. P. (2019). Single dose eradication of extensively drug resistant *Acinetobacter* spp. in a mouse model of burn infection by melittin antimicrobial peptide. *Microb. Pathog.* 127 60–69. 30513367 10.1016/j.micpath.2018.11.055

[B63] Patel S. M. Saravolatz L. D. (2006). Monotherapy versus combination therapy. *Med. Clin.* 90 1183–1195. 10.1016/j.mcna.2006.07.008 17116443

[B64] Pereira V. C. Romero L. C. Pinheiro-Hubinger L. Oliveira A. Martins K. B. Cunha M. D. L. R. D. S. D. (2020). Coagulase-negative staphylococci: a 20-year study on the antimicrobial resistance profile of blood culture isolates from a teaching hospital. *Braz. J. Infect. Dis.* 24 160–169. 10.1016/j.bjid.2020.01.003 32084346 PMC9392043

[B65] Prestinaci F. Pezzotti P. Pantosti A. (2015). Antimicrobial resistance: a global multifaceted phenomenon. *Pathog. Glob. Health* 109 309–318. 10.1179/2047773215Y.0000000030 26343252 PMC4768623

[B66] Rahimi F. Bouzari M. Katouli M. Pourshafie M. R. (2013). Antibiotic resistance pattern of methicillin resistant and methicillin sensitive *Staphylococcus aureus* isolates in Tehran, Iran. *Jundishapur J. Microbiol.* 6 144–149.

[B67] Rasoul M. Rokhsareh M. Mohammad S. M. Sajad K. Ahmadreza M. (2019). The human immune system against *Staphylococcus epidermidis*. *Crit. Rev. Immunol.* 39 151–163. 32421960 10.1615/CritRevImmunol.2019031282

[B68] Ruden S. Rieder A. Chis Ster I. Schwartz T. Mikut R. Hilpert K. (2019). Synergy pattern of short cationic antimicrobial peptides against multidrug-resistant *Pseudomonas aeruginosa*. *Front. Microbiol.* 10:2740. 10.3389/fmicb.2019.02740 31849888 PMC6901909

[B69] Sabaté Brescó M. Harris L. G. Thompson K. Stanic B. Morgenstern M. O’mahony L. (2017). Pathogenic mechanisms and host interactions in *Staphylococcus epidermidis* device-related infection. *Front. Microbiol.* 8:1401. 10.3389/fmicb.2017.01401 28824556 PMC5539136

[B70] Santos J. V. D. O. Porto A. L. F. Cavalcanti I. M. F. (2021). Potential application of combined therapy with lectins as a therapeutic strategy for the treatment of bacterial infections. *Antibiotics* 10:520. 10.3390/antibiotics10050520 34063213 PMC8147472

[B71] Sanyal D. Johnson A. George R. Cookson B. Williams A. (1991). Peritonitis due to vancomycin-resistant *Staphylococcus epidermidis*. *Lancet* 337:54. 10.1016/0140-6736(91)93375-j 1670676

[B72] Schmid A. Wolfensberger A. Nemeth J. Schreiber P. W. Sax H. Kuster S. P. (2019). Monotherapy versus combination therapy for multidrug-resistant Gram-negative infections: systematic review and meta-analysis. *Sci. Rep.* 9:15290. 10.1038/s41598-019-51711-x 31664064 PMC6821042

[B73] Schwalbe R. S. Stapleton J. T. Gilligan P. H. (1987). Emergence of vancomycin resistance in coagulase-negative staphylococci. *N. Engl. J. Med.* 316 927–931. 3821839 10.1056/NEJM198704093161507

[B74] Shang D. Liu Y. Jiang F. Ji F. Wang H. Han X. (2019). Synergistic antibacterial activity of designed Trp-containing antibacterial peptides in combination with antibiotics against multidrug-resistant *Staphylococcus epidermidis*. *Front. Microbiol.* 10:2719. 10.3389/fmicb.2019.02719 31824473 PMC6886405

[B75] Sheard D. E. O’brien-Simpson N. M. Wade J. D. Separovic F. (2019). Combating bacterial resistance by combination of antibiotics with antimicrobial peptides. *Pure Appl. Chem.* 91 199–209.

[B76] Shirvani F. Sannai Dashti A. Seifi K. (2018). Staphylococcus and linezolid resistance in Iran. *Arch. Pediatr. Infect. Dis.* 6:e12236.

[B77] Sieradzki K. Villari P. Tomasz A. (1998). Decreased susceptibilities to teicoplanin and vancomycin among coagulase-negative methicillin-resistant clinical isolates of staphylococci. *Antimicrob. Agents Chemother.* 42 100–107. 10.1128/AAC.42.1.100 9449268 PMC105463

[B78] Sridhar A. Sandeep Y. Krishnakishore C. Sriramnaveen P. Manjusha Y. Sivakumar V. (2012). Fatal poisoning by isoniazid and rifampicin. *Indian J. Nephrol.* 22 385–387. 23326053 10.4103/0971-4065.103930 PMC3544064

[B79] Światły-Błaszkiewicz A. Mrówczyńska L. Matuszewska E. Lubawy J. Urbański A. Kokot Z. J. (2020). The effect of bee venom peptides melittin, tertiapin, and apamin on the human erythrocytes ghosts: a preliminary study. *Metabolites* 10:191. 10.3390/metabo10050191 32413967 PMC7281017

[B80] Tacconelli E. Tumbarello M. De Gaetano Donati K. Bettio M. Spanu T. Leone F. (2001). Glycopeptide resistance among coagulase-negative staphylococci that cause bacteremia: epidemiological and clinical findings from a case-control study. *Clin. Infect. Dis.* 33 1628–1635. 10.1086/323676 11595984

[B81] Talebi M. Shafiee M. Sadeghi J. Moghadam N. A. Saifi M. Pourshafie M. R. (2016). Genotypic diversity of methicillin-resistant coagulase-negative staphylococci isolated from inpatients and outpatients. *Microb. Drug Resist.* 22 147–154. 10.1089/mdr.2014.0195 26248114

[B82] Tamma P. D. Cosgrove S. E. Maragakis L. L. (2012). Combination therapy for treatment of infections with gram-negative bacteria. *Clin. Microbiol. Rev.* 25 450–470. 10.1128/CMR.05041-11 22763634 PMC3416487

[B83] Traczewski M. M. Katz B. D. Steenbergen J. N. Brown S. D. (2009). Inhibitory and bactericidal activities of daptomycin, vancomycin, and teicoplanin against methicillin-resistant *Staphylococcus aureus* isolates collected from 1985 to 2007. *Antimicrob. Agents Chemother.* 53 1735–1738. 10.1128/AAC.01022-08 19223623 PMC2681544

[B84] Tuazon C. U. Miller H. (1983). Clinical and microbiologic aspects of serious infections caused by *Staphylococcus epidermidis*. *Scand. J. Infect. Dis.* 15 347–360. 10.3109/inf.1983.15.issue-4.05 6361977

[B85] Uccelletti D. Zanni E. Marcellini L. Palleschi C. Barra D. Mangoni M. L. (2010). Anti-*Pseudomonas* activity of frog skin antimicrobial peptides in a *Caenorhabditis elegans* infection model: a plausible mode of action in vitro and in vivo. *Antimicrob. Agents Chemother.* 54 3853–3860. 10.1128/AAC.00154-10 20606068 PMC2935021

[B86] Wang P.-J. Xie C.-B. Sun F.-H. Guo L.-J. Dai M. Cheng X. (2016). Molecular characteristics of methicillin-resistant *Staphylococcus epidermidis* on the abdominal skin of females before laparotomy. *Int. J. Mol. Sci.* 17:992. 10.3390/ijms17060992 27338374 PMC4926520

[B87] Wayne P. (2010). *Clinical and Laboratory Standards Institute: Performance Standards for Antimicrobial Susceptibility Testing: 20th Informational Supplement. CLSI document M100-M120.* Wayne, PA: Clinical and Laboratory Standards Institute.

[B88] Wi Y. M. Greenwood-Quaintance K. E. Brinkman C. L. Lee J. Y. Howden B. P. Patel R. (2018). Rifampicin resistance in *Staphylococcus epidermidis*: molecular characterisation and fitness cost of rpoB mutations. *Int. J. Antimicrob. Agents* 51 670–677. 10.1016/j.ijantimicag.2017.12.019 29287710

[B89] Xu W. Zhu X. Tan T. Li W. Shan A. (2014). Design of embedded-hybrid antimicrobial peptides with enhanced cell selectivity and anti-biofilm activity. *PLoS One* 9:e98935. 10.1371/journal.pone.0098935 24945359 PMC4063695

[B90] Zarghami V. Ghorbani M. Bagheri K. P. Shokrgozar M. A. (2021a). Melittin antimicrobial peptide thin layer on bone implant chitosan-antibiotic coatings and their bactericidal properties. *Mater. Chem. Phys.* 263:124432.

[B91] Zarghami V. Ghorbani M. Bagheri K. P. Shokrgozar M. A. (2021b). Prevention the formation of biofilm on orthopedic implants by melittin thin layer on chitosan/bioactive glass/vancomycin coatings. *J. Mater. Sci. Mater. Med.* 32:75. 10.1007/s10856-021-06551-5 34156547 PMC8219550

[B92] Zarrinnahad H. Mahmoodzadeh A. Hamidi M. P. Mahdavi M. Moradi A. Bagheri K. P. (2018). Apoptotic effect of melittin purified from Iranian honey bee venom on human cervical cancer HeLa cell line. *Int. J. of Pept. Res. Ther.* 24 563–570. 10.1007/s10989-017-9641-1 30416405 PMC6208649

[B93] Zasloff M. (2002). Antimicrobial peptides of multicellular organisms. *Nature* 415 389–395. 11807545 10.1038/415389a

[B94] Zasloff M. (2006). Inducing endogenous antimicrobial peptides to battle infections. *Proc. Natl. Acad. Sci. U.S.A.* 103 8913–8914. 10.1073/pnas.0603508103 16754884 PMC1482538

[B95] Zharkova M. S. Orlov D. S. Golubeva O. Y. Chakchir O. B. Eliseev I. E. Grinchuk T. M. (2019). Application of antimicrobial peptides of the innate immune system in combination with conventional antibiotics—a novel way to combat antibiotic resistance? *Front. Cell. Infect. Microbiol.* 9:128. 10.3389/fcimb.2019.00128 31114762 PMC6503114

[B96] Zimmerli W. Trampuz A. Ochsner P. E. (2004). Prosthetic-joint infections. *N. Engl. J. Med.* 351 1645–1654. 15483283 10.1056/NEJMra040181

[B97] Zimmerli W. Widmer A. F. Blatter M. Frei R. Ochsner P. E. (1998). Role of rifampin for treatment of orthopedic implant–related staphylococcal infections: a randomized controlled trial. *JAMA* 279 1537–1541. 10.1001/jama.279.19.1537 9605897

[B98] Zimmermann G. R. Lehar J. Keith C. T. (2007). Multi-target therapeutics: when the whole is greater than the sum of the parts. *Drug Discov. Today* 12 34–42. 10.1016/j.drudis.2006.11.008 17198971

[B99] Zusman O. Avni T. Leibovici L. Adler A. Friberg L. Stergiopoulou T. (2013). Systematic review and meta-analysis of in vitro synergy of polymyxins and carbapenems. *Antimicrob. Agents Chemother.* 57 5104–5111. 10.1128/AAC.01230-13 23917322 PMC3811454

